# Water-driven microbial nitrogen transformations in biological soil crusts causing atmospheric nitrous acid and nitric oxide emissions

**DOI:** 10.1038/s41396-021-01127-1

**Published:** 2021-11-11

**Authors:** S. Maier, A. M. Kratz, J. Weber, M. Prass, F. Liu, A. T. Clark, R. M. M. Abed, H. Su, Y. Cheng, T. Eickhorst, S. Fiedler, U. Pöschl, B. Weber

**Affiliations:** 1 Institute of Biology, University of Graz, Graz, Austria; 2 Multiphase Chemistry Department, Max Planck Institute for Chemistry, Mainz, Germany; 3 School of Chemical & Biomolecular Engineering, Georgia Institute of Technology, Atlanta, GA USA; 4 College of Science, Biology Department, Sultan Qaboos University, Al Khoud, Sultanate of Oman; 5 FB2 (Biology/Chemistry), University of Bremen, Bremen, Germany; 6 Institute of Geography, Johannes Gutenberg University, Mainz, Germany

**Keywords:** Soil microbiology, Biogeochemistry

## Abstract

Biological soil crusts (biocrusts) release the reactive nitrogen gases (N_r_) nitrous acid (HONO) and nitric oxide (NO) into the atmosphere, but the underlying microbial process controls have not yet been resolved. In this study, we analyzed the activity of microbial consortia relevant in N_r_ emissions during desiccation using transcriptome and proteome profiling and fluorescence in situ hybridization. We observed that < 30 min after wetting, genes encoding for all relevant nitrogen (N) cycling processes were expressed. The most abundant transcriptionally active N-transforming microorganisms in the investigated biocrusts were affiliated with *Rhodobacteraceae*, *Enterobacteriaceae*, and *Pseudomonadaceae* within the *Alpha*- and *Gammaproteobacteria*. Upon desiccation, the nitrite (NO_2_^−^) content of the biocrusts increased significantly, which was not the case when microbial activity was inhibited. Our results confirm that NO_2_^−^ is the key precursor for biocrust emissions of HONO and NO. This NO_2_^−^ accumulation likely involves two processes related to the transition from oxygen-limited to oxic conditions in the course of desiccation: (i) a differential regulation of the expression of denitrification genes; and (ii) a physiological response of ammonia-oxidizing organisms to changing oxygen conditions. Thus, our findings suggest that the activity of N-cycling microorganisms determines the process rates and overall quantity of N_r_ emissions.

## Introduction

Soils host one of the most diverse microbiomes on Earth [1] with abundances of prokaryotes (bacteria and archaea) reaching 4 – 20 × 10^9^ cm^−3^ [2]. These microorganisms are one of the major biotic drivers of the biogeochemical cycles of carbon (C), nitrogen (N), oxygen (O_2_), and sulphur [3]. Biological soil crusts (biocrusts) represent a special type of soil microbiome, colonizing the uppermost layer of the soil in arid and semi-arid ecosystems worldwide [4]. They are composed of a photoautotrophic upper layer with poikilohydric (desiccation-tolerant) organisms, such as cyanobacteria, algae, lichens, and bryophytes, and a layer below with heterotrophic microorganisms [5–8]. Biocrusts occur globally in regions with dry microclimatic conditions, such as drylands. They cover approximately 12% of the Earth’s terrestrial surface, corresponding to an area of ~18 × 10^6^ km^2^ [9, 10]. In some desert regions, up to 70% of the soil surface is covered by biocrusts [4].

Nitrogen represents an essential element for all living organisms and most of them depend on bioavailable forms, like ammonium (NH_4_^+^) and nitrate (NO_3_^−^), for growth. The availability of these reactive forms of N relies on metabolically versatile microorganisms carrying out N-transforming reactions, which are spatially and/or temporally separated [11]. Microbial transformations of N comprise the distinct processes of N fixation, assimilation into organic N, ammonification (degradation of organic N), nitrification, denitrification, and anaerobic ammonium oxidation (anammox) [11]. The N-transforming processes have considerably different fluxes, as the largest N fluxes are attributed to the interconversion of ammonia (NH_3_) and organic N (ammonification and ammonium assimilation) [11].

Nitrogen oxides (NO_x_ = NO + NO_2_) play a vital role in the formation of ozone (O_3_) in the troposphere [12]. Tropospheric O_3_ is a component of photochemical smog and a short-lived climate pollutant [13, 14]. Sources of tropospheric O_3_ are the stratosphere and in situ photochemical O_3_ formation that is dependent on the concentration of NO_x_ [12]. Major sources of NO_x_ in the troposphere are the combustion of fossil fuels, biomass burning, soil microbial activity, and lightning [15]. Nitrous acid (HONO) is one of the precursors of the hydroxyl radical (OH•). Through formation of OH•, HONO regulates the oxidation capacity of the atmosphere, which leads to the removal of most trace gases emitted by natural and anthropogenic activities [16]. Proposed atmospheric reactions for the formation of HONO involve gas phase and heterogeneous reactions of NO_x_ [17–21].

Gas exchange studies showed that the atmospherically relevant trace gases HONO and NO can be emitted from soil and biocrusts [22–30]. About 15% of the NO_x_ emissions to the atmosphere originate from soils under natural vegetation [31], with substantial amounts being emitted as NO due to the microbial processes nitrification and denitrification [32–35]. Evidence suggests that also biogenic sources of HONO exist in soil [23, 25]. It was suggested that soil nitrite (NO_2_^−^), derived from the biological processes nitrification and denitrification, can be an important source of atmospheric HONO [22] and that acidic conditions (at least at the soil surface) are needed [26, 30, 36, 37]. More recently, pure culture studies reported that ammonia-oxidizing bacteria (AOB) and archaea (AOA) are involved in the formation of HONO [23, 38], and thus also NH_3_ concentrations are likely relevant. Hydroxylamine (NH_2_OH) has been identified as a further precursor for the formation of HONO [38]. Peak fluxes of HONO were also related to the abundance of nitrifying microbes in agricultural and urban soil and stable isotope tracer experiments demonstrated the microbial oxidation of NH_4_^+^ to HONO [39]. Microbial NO_3_^−^ reduction (denitrification) was described as another pathway for HONO production via soil NO_2_^−^ in aerobic, agricultural soils [40]. For O_2_-limited microsites in wet soils, HONO emissions were attributed to NO_2_^−^, formed by microbial NO_3_^−^ reduction [41].

In multiple studies, NO and HONO emissions were strongly related to the water content and emissions from drying soil, soil bacteria, and biocrusts were minimum at high moisture, i.e., 100% water holding capacity (WHC) [22, 23, 25, 26]. With progressive moisture reduction, HONO and NO emissions started to rise, reaching maximum values around 20–30% WHC. This study aims to elucidate the microbial processes involved in HONO and NO production. We combined continuous, dynamic NO and HONO flux measurements with fluorescence in situ hybridization (FISH), and transcriptome (microarray) and proteome profiling of drying biocrusts. For this, biocrust samples were wetted to full WHC and continuous flux measurements were conducted over the desiccation cycle. At characteristic stages, i.e., at full WHC, before the emission maximum, and shortly before complete drying, the flux measurements were stopped to analyze the activity and abundance of microorganisms. The functional gene microarray (FGA) was applied to identify pathways and the taxonomic identity of microorganisms involved in N transformations. Fluorescence in situ hybridization was used to study the spatial distribution and abundance of microbes that contribute to the N cycle in biocrusts. In addition, the NO_2_^−^ and NO_3_^−^ content of the samples was analyzed before and after a desiccation cycle, and in a parallel approach the relevance of biotic processes was analyzed by suppressing the biotic activity by methyl iodide (CH_3_I) treatment of the samples. With this combined approach, we aimed to (i) analyze the response of microorganisms to wetting following a severe dry period, (ii) identify the organisms responsible for the different N-transforming processes, and (iii) investigate the temporal connection between microbial metabolic activity and the emissions of HONO and NO. The results of this study should help to elucidate the underlying microbial processes causing the release of NO and HONO emissions by biocrusts.

## Material and methods

### Study area and sampling

Samples were collected within the Succulent Karoo biome (Fig. S1), stretching along the Atlantic coast of southwestern Namibia and South Africa into the dry intermountain valleys and basins within the Cape Fynbos biome [42]. The area is characterized by a semi-arid climate with an annual precipitation of ~131 mm occurring during the winter months (July and August) and a mean air temperature of 19.4 °C [43, 44]. More information on the study area is available in Haarmeyer et al. [43] and additional information on the sampling procedure is available in the Supplementary Material 1.

### Overall experimental setup

**Fig. 1:**
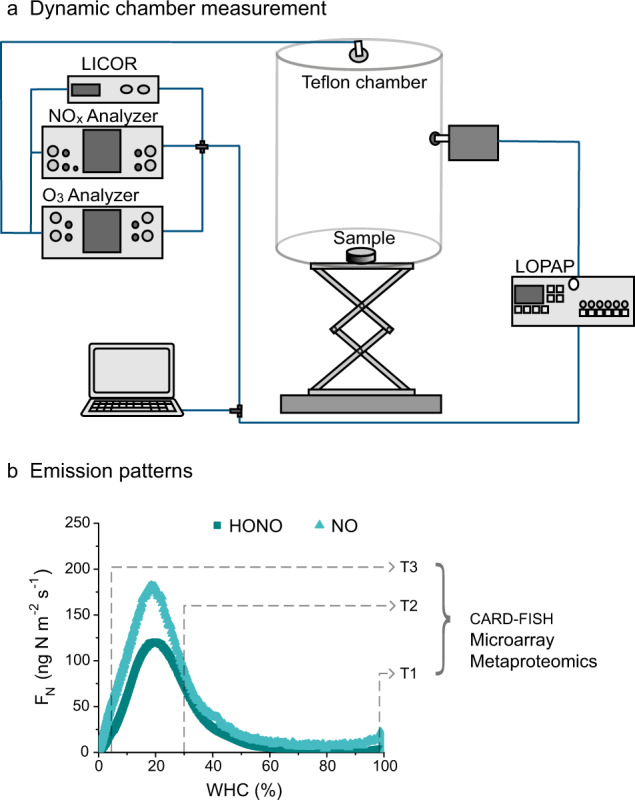
Overview of the experimental setup. **a** Dynamic chamber system for measurements of reactive nitrogen (N_r_) fluxes; (**b**) Representative emission curves/patterns with three stages during a desiccation cycle (T1: early wetting; ~99% mean water holding capacity (WHC); T2: intermediate drying; ~30% mean WHC; T3: late drying; ~4% mean WHC). At each of the stages, the dynamic chamber measurements were stopped and microarray, metaproteomic, and CARD FISH analyses were conducted (the latter only at T1 and T2).

Dynamic chamber measurements were conducted at 25 °C in the dark (Fig. 1a). Biocrust samples were wetted with sterile, artificial rain water [45] up to full WHC. At three characteristic stages during one desiccation cycle (wetting and subsequent drying), the experiment was stopped, samples (*n* = 3) were removed from the chamber, and used for further analyses (Fig. 1b). The first stage was 20–30 min after wetting (T1; mean ~99% WHC; Table S1a), the second at increasing emissions prior to maximum N_r_ fluxes (T2; mean ~30% WHC; 3.4–5 h after wetting; Table S1a), and the third shortly before complete desiccation (T3; mean ~4% WHC; 6.3–8 h after wetting; Table S1a). One part of the replicate of each desiccation stage was used for RNA extraction and subsequent microarray analysis (GeoChip) to detect the actively transcribed genes involved in biogeochemical cycling of N and a second part was used for mass spectrometry-based metaproteomics. A third part was used to quantify the number of bacteria, archaea and nitrite-oxidizing bacteria (NOB) by means of CARD-FISH (Catalyzed Reporter Deposition Fluorescence in situ Hybridization), but here only samples at T1 and T2 were investigated (Fig. 1b). In addition, we analyzed the NO_2_^−^ and NO_3_^−^ contents before (Pre) and after a desiccation cycle (Post) and the relevance of biotic processes was tested by suppressing the biotic activity by means of a CH_3_I treatment. Furthermore, the O_2_ concentration was measured using microsensors at T1 and T2. For details on soil analyses and local measurements of O_2_, see Supplementary Material 1.

### Dynamic chamber measurements

The measurement of HONO and NO emissions was conducted using a dynamic Teflon chamber (Fig. 1a; Supplementary Material 1). The emissions and mixing ratios of NO, HONO, NO_2_ (nitrogen dioxide) and H_2_O were measured at the outlet of the chamber (volume of 0.047 m^3^). NO and NO_2_ were analyzed with a gas chemiluminescence detector equipped with a blue light converter (Model 42 C, Thermo Electron Corporation, Waltham, Massachusetts, USA). HONO was detected spectrophotometrically using a long path absorption photometer (LOPAP, QUMA Elektronik & Analytik GmbH, Wuppertal, Germany). Full details of the chamber measurements are given in the Supplementary Material 1.

### Quantification of bacterial, archaeal and NOB populations in soil samples by CARD-FISH

CARD-FISH allows the quantification of soil microorganisms and the analysis of spatial and temporal dynamics of native microbial populations [46]. We utilized this method to obtain information on the cell number of archaeal and bacterial cells and NOB at T1 and T2 in different biocrust layers during the desiccation cycle.

Samples of the upper photoautotrophic (0– ~0.5 mm depth) and the lower heterotrophic layer (~0.5–9 mm depth) were analyzed at T1 and T2 to study the temporal and spatial distribution of the microbial populations. For details on the CARD-FISH procedure, see the Supplementary Material 1.

### GeoChip functional gene microarray

We applied the FGA GeoChip 5.0, manufactured by Agilent Technologies (Santa Clara, CA, USA), to analyze the microbial transcriptional activity of genes involved in N-cycling processes within biocrusts and its change over the course of a desiccation cycle [47, 48]. As the probes on GeoChip are based on gene sequences from pure cultures or from environmental sequences of known taxonomic groups, the hybridization data enables an assignment of the metabolic capabilities to bacterial and archaeal groups. For information on RNA extraction, cDNA synthesis and data processing see Supplementary Material 1.

### Mass spectrometry-based metaproteomics

Mass spectrometry-based metaproteomics was used to determine which proteins were produced during the three characteristic stages of desiccation. The study of the entire set of proteins, resulting from cellular processes of microorganisms within their natural environment, provides insights into the microbial activity patterns [49]. Detailed information on the analytical procedure is available in the Supplementary Material 1.

## Results

### N_r_ emissions and mineral nitrogen content of biocrusts

**Fig. 2:**
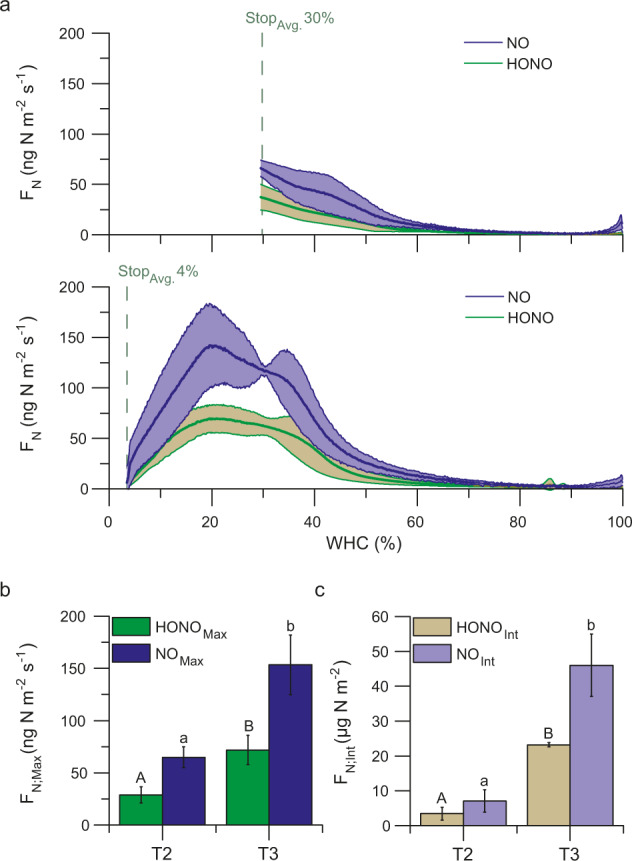
Reactive nitrogen emissions along a desiccation cycle. **a** Average HONO and NO emission curves along a desiccation cycle of cyanobacteria-dominated biocrusts from South Africa (25 °C and in the dark) stopped at different stages during desiccation (T2, T3). Lines indicate the mean fluxes and shaded areas the standard deviation; (**b**) Comparison of maximum HONO and NO as well as (**c**) integrated HONO and NO emissions of the measurements stopped at different stages during desiccation (T2, T3). At T2 the samples had a mean WHC of ~30% and at T3 ~4%; *n* = 3.

Over the course of the flux measurements, the highest HONO and NO emissions were observed at ~20% WHC (mean max. HONO: 71.82 ± 14.0 ng N m^−2^ s^−1^; mean max. NO: 153.49 ± 28.63 ng N m^−2^ s^−1^). The maximum HONO and NO values at T2 were significantly lower as compared to T3 (Fig. 2; HONO_Max_: DF = 4, *t* value = −4.64, *p* = 0.01; NO_Max_: DF = 4, *t* value = −2.82, *p* = 0.048; Table S1b), since the measurements were stopped before the maximum emissions were reached. Also the integral emissions of HONO and NO were higher for the samples stopped at T3 as compared to those stopped at T2 (HONO_Int_: DF = 4, *t* value = −17.69, *p* = 6.00 × 10^−5^; NO_Int_: DF = 4, *t* value = −7.08, *p* = 0.002; Fig. 2; Table S1b).

**Fig. 3:**
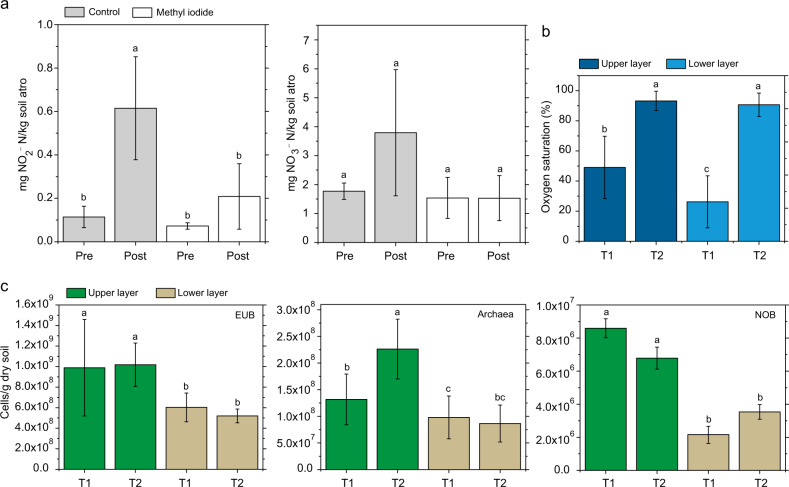
Nutrient content, oxygen saturation, and cell numbers at varying sampling times and in different strata. **a** Nitrite and nitrate contents of control and methyl iodide (CH_3_I) treated samples before (pre) and after (post) a desiccation cycle. Error bars indicate standard deviation and different letters indicate significant differences (one-way ANOVA and post hoc tests, *p* < 0.01, *n* = 3); atro = absolutely dry. **b** Average oxygen saturation (%) obtained at T1 (99% WHC) and T2 (30% WHC) in the upper (photoautotrophic layer; 0–400 µm) and lower layer (heterotrophic layer; > 400–3000 µm) within biocrust samples. Bars represent mean values, error bars indicate standard deviation (*n* = 8). Results of two-way repeated measures ANOVA and post hoc tests (*p* < 0.05) are given as letters on top of bars. **c** Cell numbers of CARD-FISH-stained bacteria (EUB), archaea and nitrite-oxidizing bacteria (NOB) per gram of soil detected at stage 1 and 2 during desiccation in the upper layer and lower layer of the biocrust. Bars represent mean values of 1000 cell counts on filter sections per biocrust samples. Error bars indicate standard deviation (*n* = 3). Results of Bayesian hierarchical model fitted to account for pseudoreplication (posterior probability < 0.05) are given as letters on top of bars.

Prior to full desiccation cycles, the NO_2_^−^-N and NO_3_^−^-N contents of biocrusts were similar in control and CH_3_I treated samples (Fig. 3a). After a desiccation cycle, the NO_2_^−^-N and NO_3_^−^-N contents of the control samples tended to be higher, with a statistically significant increase registered for the NO_2_^−^-N content (*q* = 6.607, *p* = 0.007, *n* = 3; Fig. 3a; Table S1c). Samples treated with CH_3_I showed low NO_2_^−^-N and NO_3_^−^-N contents in a similar range as the samples analysed before the desiccation experiment (Fig. 3a).

### Local measurements of O_2_

The average O_2_ saturation varied depending on the water content of the biocrust. At T1 (~99% WHC), O_2_ was limited in the photoautotrophic and more strongly in the heterotrophic layer. At T2 (~30% WHC) we detected high O_2_ concentrations throughout the biocrust (Fig. 3b).

### Spatial and temporal distribution and abundance of microbial populations

Cell numbers of bacteria and archaea were highest in the upper layer at T2 showing values of 1.0 × 10^9^ and 2.3 × 10^8^ cells per gram of soil, respectively (Fig. 3c). The number of bacterial cells per gram of soil was 4.5–7.5 times higher than that of archaea and about 1% of the bacteria belonged to the group of NOB (Fig. 3c). Bacterial cell numbers and NOB cell numbers were significantly higher in the upper layer compared to the one below (Bayesian hierarchical model, *p* < 0.05, *n* = 3). In addition, the upper biocrust layer showed significantly higher archaeal counts compared to the lower layer for T1 and T2 (Bayesian hierarchical model, *p* < 0.05, *n* = 3). For bacterial cell numbers and NOB, no statistically significant differences were observed between T1 and T2. On the contrary, the number of archaea in the upper biocrust layer increased significantly by 72% from T1 to T2 (Bayesian hierarchical model, *p* < 0.05, *n* = 3). In relation to all prokaryotic cells, the percentage of archaea increased from 11.7% at T1 to 18.2% at T2, whereas in the lower biocrust layer no clear change was detectable. We were unable to detect signals when using the probe Nso1225 for AOB (Table S2).

### Microbes involved in nitrogen-transforming processes

**Fig. 4:**
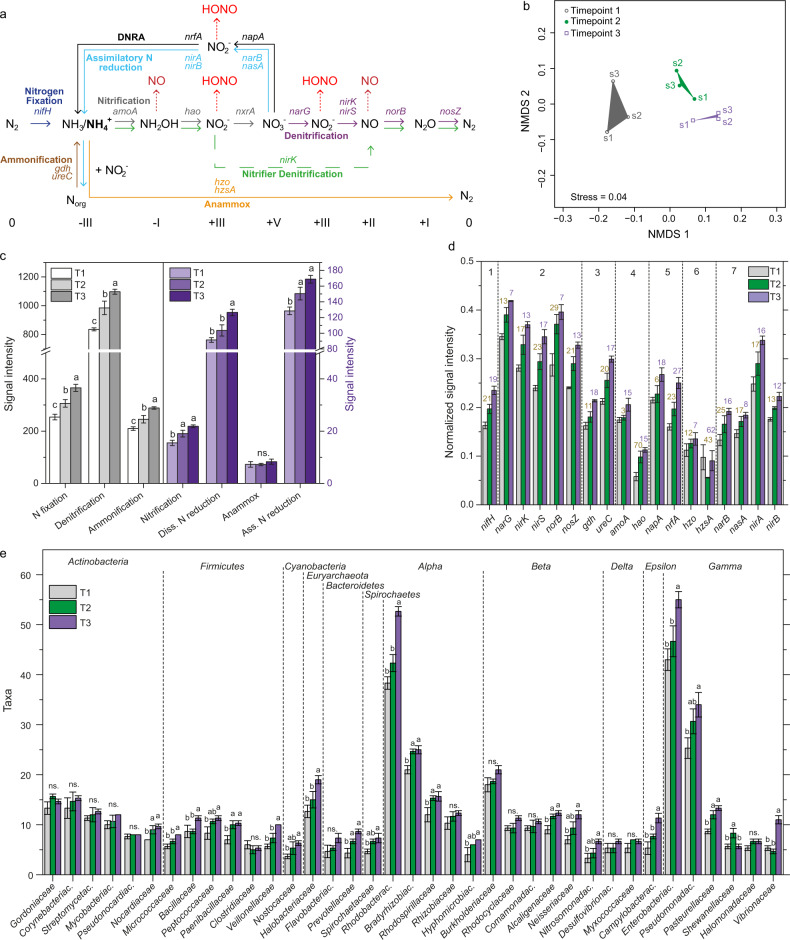
Schematic representation of the N cycle and analyses of data from the functional gene microarray. **a** Biogeochemical N cycle: N-transforming processes and encoding genes catalyzing N-cycling reactions are shown (**b**–**e**) Functional gene microarray. **b** Nonmetric multidimensional scaling (NMDS) plot of Bray-Curtis similarities for the functional gene profiles identified at different stages during desiccation (**c**, **d**) Signal intensity per N-transforming process/gene detected at different stages of the desiccation cycle of biocrusts. Mean values of three replicates per stage during desiccation were plotted with the standard deviation. Differences between stages of desiccation were tested with a one-way ANOVA and labelled with lowercase letters. In (**d**) the signal intensity was normalized by the number of probes for each gene on the array. Numbers above bars at T2 show percentual increase between T1 and T2, those above bars at T3 show percentual increase between T2 and T3.1: N fixation; 2: Denitrification; 3: Ammonification; 4: Nitrification; 5: Dissimilatory N reduction; 6: Anammox; 7: Assimilatory N reduction (**e**) N-transforming microorganisms within the phyla *Actinobacteria*, *Firmicutes*, *Cyanobacteria*, *Bacteroidetes*, *Spirochaetes*, *Euryarchaeota* and in families within the phylum *Proteobacteria*. Families with >5 taxa were plotted. For full list of identified organisms, see Table S7.

Hybridization of cDNA was achieved for an average of 27.6% of the 57,000 probes (Table S3), showing that the procedure worked properly [48]. 13% of the probes with positive hybridization signals could be allocated to the category of N-transforming processes (Table S3). More explicitly, mRNA transcripts of genes encoding enzymes for N fixation, ammonification, nitrification, denitrification, anammox, dissimilatory nitrate reduction to ammonium (DNRA), and assimilatory nitrate reduction were detected (Fig. 4a). Hierarchical cluster analysis (Figs. S2, S3) and a nonmetric multidimensional scaling illustrated that the gene expression profiles differed clearly between the three stages during desiccation (Fig. 4b), with samples taken during the same stage being more similar than those taken during different stages. An analysis of similarity (ANOSIM) test supported the conclusion that the metabolic potential was statistically different between the three stages of desiccation (ANOSIM *r* = 0.876, *p* = 0.003, *n* = 3, permutation = 9999). The overlap of the detected probes between the samples is shown in Table S4 and S5.

Biocrust samples comprised a reservoir of transcripts (mRNA) involved in the major pathways of the N cycle, suggesting microbial contributions to the different N transformations (Fig. 4c). The number of mRNAs detected differed widely, ranging from 0 to ~400, with the highest numbers observed for *nifH* and particularly low numbers for genes encoding enzymes for nitrification and anammox (Fig. S4). During the desiccation cycle, we observed a rapid recovery of the metabolism, as at T1, 20–30 min after wetting, genes involved in all major N-transforming processes were already induced (Fig. 4c, d). For most processes, the mRNA levels of functional genes showed an increase with time, except for anammox (Fig. 4c). For N fixation (*nifH*) and ammonification (*ureC*), there was a rather homogeneous increase in gene expression. Denitrification and assimilatory N reduction showed a stronger increase during the first phase (from T1 to T2), and DNRA during the second phase of the desiccation cycle (from T2 to T3) (Fig. 4d).

The number of N-transforming species increased significantly from T1 to T2 (320 and 366 species) and from T2 to T3 (410 species; Fig. 4e). Also on higher taxonomic levels, this overall increase could be observed (Table S6). A large portion of the metabolically active bacteria during the desiccation cycle belonged to *Alpha*- and *Gammaproteobacteria*. The number of taxa involved in N-cycling in biocrusts was quite similar for a large range of bacterial families (ranging between ~5 and ~15) but there were also some families that provided large numbers of taxa, as e.g., *Rhodobacteraceae*, *Bradyrhizobiaceae*, *Enterobacteriaceae*, and *Pseudomonadaceae* (Fig. 4e).

**Fig. 5:**
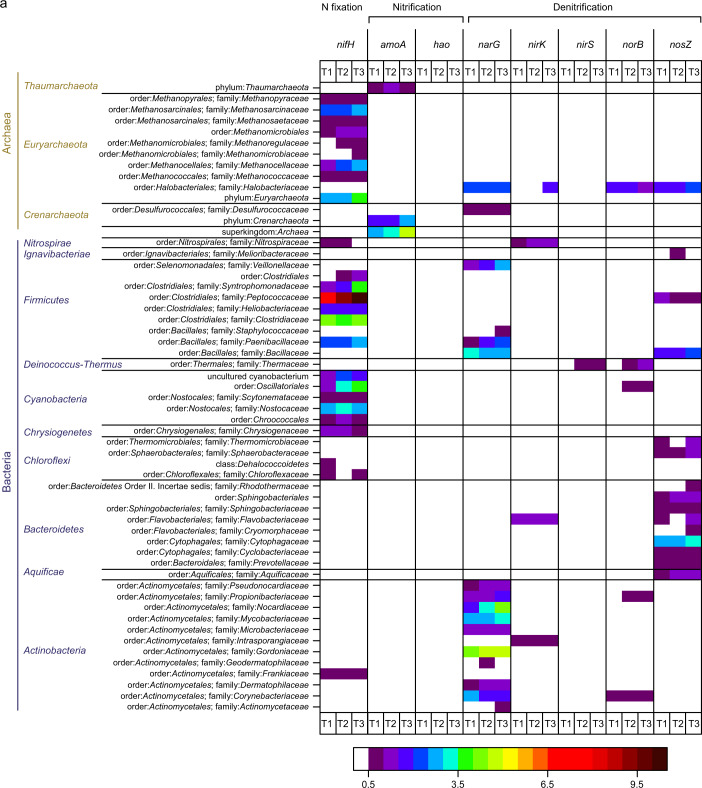

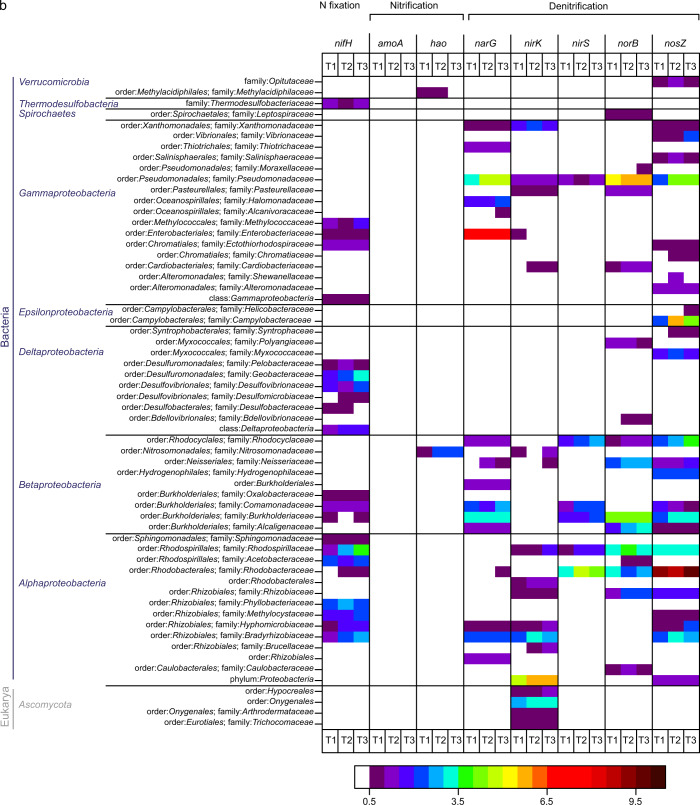
Functional gene microarray. **a**, **b** Taxa-function relationship for N-cycling genes. Mean normalized signal intensity is shown. White color indicates non-detected signal, while intensity of positive signals is indicated from blue (lower signal intensities) to red color (higher signal intensities). Unclassified bacteria are not shown.

The *nifH* genes, indicative of diazotrophic ability, were phylogenetically widespread, but particularly abundant among *Euryarchaeota*, *Firmicutes*, *Cyanobacteria*, and *Alphaproteobacteria* (Fig. 5). The onset of N fixation required more extended periods of recovery in *Methanoregulaceae*, *Methanomicrobiaceae*, *Clostridiales*, *Desulfomicrobiaceae*, and *Rhodobacteraceae* as compared to the other detected diazotrophs (Figs. 5, S5). Potential nitrification activity was attributed to the domain of *Archaea* and the class of *Betaproteobacteria*. The most abundant denitrifiers were concentrated within the phyla *Bacteroidetes*, *Actinobacteria*, and *Proteobacteria*. Ammonification genes were widely spread across the domains *Archaea*, *Bacteria*, and *Eukarya*, and highest values of signal intensity (for *ureC*), increasing during the desiccation cycle, were observed in organisms affiliated with *Rhodobacteraceae*, *Streptomycetaceae*, and *Enterobacteriaceae* (Fig. S6).

**Fig. 6:**
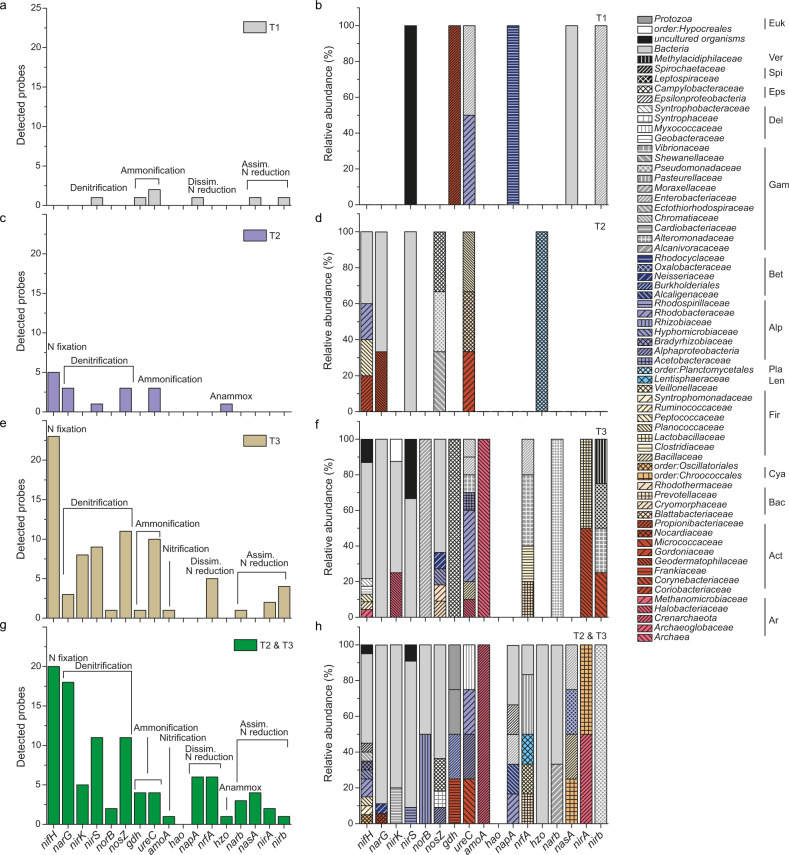
Functional gene microarray. **a**, **c**, **e** Number of probes per gene only detected at **a** T1, **c** T2, **e** T3, and **g** T2 and T3. **b**, **d**, **f**, **h** Taxonomic groups of the detected probes per gene at the family level detected at **b** T1, **d** T2, **f** T3, and **h** T2 and T3. Ar: *Archaea*; Act: *Actinobacteria*; Bac: *Bacteroidetes*; Cya: *Cyanobacteria*; Euk: *Eukarya*; Fir: *Firmicutes*; Len: *Lentisphaerae*; Pla: *Planctomycetes*; Alp: *Alphaproteobacteria*; Bet: *Betaproteobacteria*; Gam: *Gammaproteobacteria*; Del: *Deltaproteobacteria*; Eps: *Epsilonproteobacteria*; Spi: *Spirochaetes*; Ver: *Verrucomicrobia*. N fixation: nitrogen fixation; dissim. N reduction: dissimilatory nitrate reduction; assim. N reduction: assimilatory nitrate reduction.

The microarray profiles were compared to identify changes of gene expression over the course of the desiccation cycle. Of the 2569 tested probes, the transcript levels of 221 probes changed significantly during desiccation (Fig. 6a, c, e, g). Of these 221 probes, some were expressed in several stages, others were only detected at one point in time (expressed exclusively). Along with the increasing number of detected probes, 7 (3.2%), 16 (7.2%), and 79 probes (35.7%) were only detected at T1, T2, and T3, respectively (Fig. 6a, c, e). The analysis also revealed a strong overlap between the genes induced during T2 and T3, as 99 probes (44.8%) were detected at T2 and T3 but not at T1 (Fig. 6g). Among genes, of which the expression was initiated at T3 (Fig. 6e) or T2 and T3 (Fig. 6g), there were probes coding for all studied N-transforming processes. In contrast, no genes coding for N fixation, nitrification, and anammox were expressed exclusively at T1, and those coding for nitrification and N reduction processes were not expressed exclusively at T2 (Fig. 6a, c). Different bacterial families became active and the number of families tended to increase with progressing desiccation (Fig. 6b, d, f, h). Generally, at T1 there were only few uniquely detected bacterial families, whereas towards T2 and T3 the taxonomic diversity showed a substantial increase.

### Proteomic response to desiccation stress

A total of 60, 42, and 44 protein groups were identified for T1, T2, and T3, respectively. There was a greater overlap in shared proteins between T1 and T2 (18.6%) as compared to T2 and T3 (3.4%), whereas 6.4% of the proteins were shared between all three stages (Fig. S7a). When considering the leading proteins, with the highest number of identified peptides, 88% of the identified proteins were assigned to bacterial species (mainly *Cyanobacteria* and *Alphaproteobacteria*), while the remaining proteins originated from eukaryotes (fungi and protists) (Supplementary Material 2). Proteins were grouped into categories based on gene ontology terms. The majority of the identified protein groups was related to ATP synthesis, photosynthesis, protein biosynthesis, and stress response (Fig. S7b–e). Some categories, such as ATP synthesis, carbohydrate metabolism, and protein biosynthesis were less represented in the samples of T3 compared to T1 and T2, whereas the number of protein groups involved in fatty acid metabolism and photosynthesis was higher at T3. Protein groups linked to stress response, such as chaperones that are produced during stress conditions e.g., desiccation, occurred in larger numbers at T1 and T3 compared to T2 (Fig. S7). The identified proteins and their taxonomic classifications are given in the Supplementary Material 2. Proteins associated with N transformations could not be identified.

## Discussion

### Effect of soil moisture and mineral nitrogen content on N_r_ emissions

At high water contents, nearly no HONO and NO emissions could be determined (Fig. 2a). Starting between 70% and 80% WHC, emissions increased, first in a linear and then in an exponential manner. Maximum fluxes of 71.8 ± 14 ng m^−2^ s^−1^ of HONO-N and 153.5 ± 28.6 ng m^−2^ s^−1^ of NO-N were observed at ~20% WHC (Fig. 2a). Similar emission ranges of HONO-N and NO-N from soil, ranging between 2–260 ng m^−2^ s^−1^ and 2–135 ng m^−2^ s^−1^, respectively, at WHC values between ~0% and 40% have been previously reported [23]. Arid and semi-arid soils were observed to emit NO-N ranging from 1.2 to 142 ng m^−2^ s^−1^ at optimum soil moisture conditions [50]. In an earlier study, biocrusts from South Africa released similar NO and HONO emission fluxes with maximum values at 20–25% WHC [25]. Maximum HONO-N and NO-N fluxes of 27.1 ± 16.1 and 26.5 ± 15.9 ng m^−2^ s^−1^, respectively, for cyanobacteria-dominated biocrusts from the Mediterranean island of Cyprus were measured at 17–33% WHC [26]. Thus, the emission patterns and rates observed in this study are consistent with previously reported data.

Measurements of soil N revealed a significantly higher NO_2_^−^-N content after a full desiccation cycle, and the NO_3_^−^-N content showed a similar trend, whereas for CH_3_I treated biocrust samples the desiccation cycle had no substantial effect on NO_2_^−^ and NO_3_^−^ production, most likely due to suppressed microbial activity (Fig. 3a). These results are in line with findings of a previous study, reporting a continuous NO_2_^−^ accumulation for a dryland soil over the course of drying [51]. Su et al. [22] were one of the first who described that soil NO_2_^−^ can serve as a strong source of atmospheric HONO and that fertilized soils with high HONO* and low pH (given the same NO_2_^−^ content) appear to be particularly strong sources of HONO and OH•. The fluxes of HONO and NO have been shown to decrease by ~75% for soil treated with CH_3_I compared to untreated soil [23], which was concomitant with a 92% decrease in the ATP content, used as an indicator for microbial activity. The addition of the nitrification inhibitor thiourea (CH_4_N_2_S) blocked the oxidation of NH_4_^+^ to NO_2_^−^ and NO_3_^−^ in soils and caused decreased HONO emissions from upland soils [29]. Similarly, NO and HONO emissions of autoclaved dark cyanobacteria-dominated biocrusts from South Africa clearly declined as a result of sterilization [25]. Thus, our results suggest, coherently with previous studies, that microorganisms are responsible for the production of NO_2_^−^ and increased emission rates of NO and HONO.

The observed accumulation of NO_2_^−^ (and a similar trend for NO_3_^−^) in untreated biocrust samples during a desiccation cycle (Fig. 3a) could be caused by an imbalance in the rates of its production and consumption during localized N cycling processes, i.e., nitrification, denitrification, assimilatory N reduction, DNRA, and anammox occurring simultaneously in microenvironments. For NO_2_^−^ to accumulate during nitrification, this would mean that the NH_3_-oxidizing activity is stimulated and/or the NO_2_^−^-oxidizing (and NO_3_^−^ reducing) activity is limited relative to the NH_3_-oxidizing potential. This can be caused by an inhibition of NO_2_^−^-oxidizing bacteria by high soil pH and/or high concentration of free NH_3_ and HNO_2_ during nitrification [52, 53]. In non-cropped Oregon soils, NO_2_^−^ accumulation caused by AOA and AOB occurred within 6 h after wetting to field capacity and persisted over 48 h, whereas NO_3_^−^ accumulation increased over time. Upon the addition of *Nitrobacter vulgaris*, a NOB, to the soil slurry, NO_2_^−^ accumulation was inhibited and NO_3_^−^ accumulation increased, while the overall rate of nitrification was unaffected [54]. It is known that AOB retain their ammonia oxidation capacity after long-term NH_4_^+^ starvation [55–58], whereas *Nitrobacter winogradskyi*, belonging to NOB, lost 80% of the NO_2_^−^-oxidizing capacity after NO_2_^−^ deprivation for six days. Accumulation of NO_2_^−^ could also happen during denitrification, and here several factors are involved in the process. Differential repression of nitrite and nitrate reductases, the competition for electron donors between nitrite and nitrate reductases, as well as pH values, O_2_ and NO_3_^−^ concentrations, and utilisable C influence a potential NO_2_^−^ accumulation during denitrification [59, 60]. Such a difference in the overall process rates may explain a NO_2_^−^ (and NO_3_^−^) accumulation in soil and biocrusts over the course of a desiccation cycle.

### Nitrogen-transforming microorganisms

The potential for N-transforming processes, represented by mRNA transcripts [47, 48], increased over the course of the desiccation cycle (Figs. 4c, d; S4). The *nifH* genes, indicative of diazotrophic ability, were widespread, but particularly abundant among *Euryarchaeota*, *Cyanobacteria*, *Firmicutes*, and *Alphaproteobacteria* (Fig. 5). These results illustrate, that N fixation in biocrusts was not solely accomplished by diazotrophic cyanobacteria, but also by various other bacteria and archaea, corroborating previous observations [61, 62].

Potential nitrification was attributed to *Archaea* and *Betaproteobacteria*, but the number of probes with positive hybridization signals and the overall signal intensity was relatively low when compared to the other processes, as N fixation and denitrification. Similarly low signal intensities were observed in a previous study [63, 64], where the same method (Geochip 5.0) was applied, whereas in other studies on biocrusts the potential activity of nitrification enzymes was similarly high as that of N fixation and denitrification enzymes [65]. Thus, it seems that the probes for key nitrification genes of the FGA do not cover a significant part of potential nitrifiers in biocrusts. Aerobic ammonia-oxidizing organisms occur in phylogenetically coherent groups within the *Beta*- but also *Gammaproteobacteria* (genera *Nitrosospira*, *Nitrosomonas*, and *Nitrosococcus*) and in the *Archaea* [66, 67]. Furthermore, for nitrifiers it has been shown, that also small populations can be very important in N transformations, due to their high substrate requirements, resulting in large quantities of intermediates and the end product NO_3_^−^ [68].

Denitrifiers were broadly distributed across soil bacteria with *Bacteroidetes*, *Actinobacteria*, and *Proteobacteria* as most abundant phyla (Fig. 5). This is in line with the literature, describing representatives of >60 genera of the domains of *Bacteria* and *Archaea*, as well as some eukaryotes as denitrifiers [66, 67]. A significant fraction of genes involved in denitrification and N fixation were assigned to unclassified bacteria, which was also observed in a previous study [64].

Proteins from genes associated with N transformations could not be detected with the current approach (Fig. S7). This could be caused by a general problem impeding proteome approaches, like (i) difficulty to detect low-abundance proteins, as their detection is obscured by highly expressed proteins (e.g., ATP synthases), (ii) difficulty to identify proteins within complex environments such as soils, and (iii) poor protein extraction efficiency from soil [69–72]. Nevertheless, at T1 and T3, more chaperones were detected than at T2, and more proteins involved in the fatty acid metabolism occurred at T3 than at T2, presumably reflecting the need for the community to cope with shifts in moisture conditions [73–75]. Bacteria exposed to fluctuations in water status are capable of modifying the cell membrane. The upregulation of shock-response genes, including those encoding molecular chaperones are associated with xerotolerance in bacteria [74]. The observed drop in ATP and protein biosynthesis at T3 might indicate that microorganisms enter a reversible form of dormancy, a common response of bacteria to abiotic stress [74, 76].

### Timing and water dependency of microbial activity

In the current study, we observed that already shortly after wetting of the biocrusts, the conditions became favourable for microbial metabolism. As early as 30 min upon wetting, significant amounts of mRNA transcripts of genes involved in the major pathways of the N cycle, including N fixation, denitrification, and nitrification, were detectable, suggesting that the organisms and possibly enzyme activity persist during dry periods in soil (Fig. 4c, d).

We observed an increase in the number of microbial taxa becoming active along a desiccation cycle as well as a sequence of different microbial taxa contributing to various N-transforming processes (Figs. 4e, 5, 6). These results are consistent with previous studies investigating the response of soil bacteria to desiccation events [77–79]. Within two hours after rewetting, the soil bacterial community (rRNA-based) returned to pre-dry community composition [79]. Furthermore, upon rewetting, transcript copies of bacterial *rpoB* genes encoding a subunit of bacterial RNA polymerase increased, indicating rapid resumption of transcriptional activity [79]. In these studies, bacteria responding rapidly to wetting have been linked to CO_2_ emissions. Most bacteria displayed their highest activity 15 min to one hour after wetting [78]. Such a “respiration burst” and a quick bacterial response upon wetting events has also been described for biocrust environments [80–82]. Denitrification in cyanobacteria-dominated crusts from the Sultanate of Oman started within two hours under wet and anaerobic conditions [83].

Already shortly after rewetting (at T1), AOB and AOA were active, suggesting that a longer dry spell (likely accompanied by a starvation of the microorganisms) did not have a major effect on the ammonia oxidation capacity (Figs. 4d, 5). Similar observations were made in a previous work, showing an increase in transcripts of the bacterial *amoA* genes within one hour and of archaeal *amoA* within nine hours after water addition [55]. However, one also has to bear in mind that a diffusion limitation of O_2_ at high water contents has a profound effect on the physiology of ammonia-oxidizing microorganisms [37]. Under oxic conditions, nitrification occurs in an unhampered way through the activity of AOB/AOA and NOB [84]. Under O_2_ limitation, however, AOB can undergo physiological changes, e.g. by inducing nitrifier denitrification, which may cause a release of NO. Besides the key enzyme ammonia monooxygenase (AMO), most AOB have a nitrite reductase, NirK, which is necessary for efficient ammonia oxidation at atmospheric O_2_ levels and involved but not essential for NO production during nitrifier denitrification [84]. The transcription of *nirk* (nitrite reductase-encoding gene) was lower during O_2_-limited conditions in *Nitrosomonadaceae* and was upregulated at T3, likely as a result of higher NO_2_^−^ concentrations, indicating efficient ammonia oxidation (Fig. 5). These gene expression patterns are consistent with previous observations [84].

The expression of archaeal *amoA* and the increased number of archaea, quantified by means of CARD-FISH at T2 (Fig. 3c), could contribute to nitrite accumulation around T2. The increase in cell numbers is likely caused by growth, as archaea are supposed to have rapid reproduction times (often below 1 h) [85]. Molecular studies indicate that AOA often outnumber nitrifying bacteria in marine [86] and terrestrial ecosystems [87, 88], and there are indications that AOA also contribute to nitrification in seasonally dry ecosystems [89, 90].

Besides nitrification, also microbial NO_3_^−^ reduction processes may be relevant. We found genes encoding denitrification, assimilatory N reduction, and DNRA in substantial quantities, while anammox was hardly observed (Fig. 4c), in coherence to former biocrust studies [64, 91]. The expression of *narG*, *nirK*, *nirS*, *norB*, and *nosZ* was considerably higher than for other functional genes, suggesting that denitrification may play a prominent role in reduction processes, and thus in the HONO and NO emissions from biocrusts. There was a substantial increase in *narG*, *nirk*, *nirS*, *norB*, and *nosZ* expression between T1 and T2, which was by far lower between T2 and T3, indicating that denitrifier activity may have been particularly strong before maximum HONO- and NO-emissions (Fig. 4d). In addition, the emission of HONO and NO has been suggested to be linked to a strongly localized pronounced drop in pH, and denitrifiers are hampered by O_2_ availability over the course of drying [37]. The synthesis of denitrification enzymes has been shown to be controlled by O_2_ levels, with NO_2_^−^ reduction as the most sensitive step during denitrification [52, 92]. Our gene expression analyses showed that *narG* expression (responsible for NO_3_^−^reduction) within the families *Nocardiaceae*, *Pseudomonadaceae*, *Enterobacteriaceae*, and *Burkholderiaceae* was considerably higher at T3 than T2 or remained more or less unchanged at T2 and T3 for *Gordoniaceae* (Fig. 5). On the contrary, expression levels of the *nirK* and *nirS* genes (responsible for NO_2_^−^ reduction) were lower than for *narG* (*Pseudomonadaceae*, *Burkholderiaceae*) or no transcription could be detected during drying (e.g., *Nocardiaceae*, *Enterobacteriaceae*, *Gordoniaceae*, *Desulfurococcaceae*, *Veillonellaceae*, *Paenibacillaceae*, *Bacillaceae*, *Pseudonocardiaceae*, *Propionibacteriaceae*, *Mycobacteriaceae*) (Fig. 5). These results suggest that differences in the expression of nitrate and nitrite reductases during the shift from O_2_-limited to aerobic conditions in the course of drying as well as high NO_3_^−^-N contents likely contribute to accumulation of NO_2_^−^, which is relevant for HONO and NO emission fluxes from biocrusts and likely also from soils. In other studies, high concentrations of NO_3_^−^, free NH_3_, and HNO_2_ were found to have an inhibitory effect on NO_2_^−^reduction [92]. In the literature, the role of denitrification in biocrusts has been considered differently, ranging from largely irrelevant [91] to highly relevant [64, 83]. For HONO emissions from agricultural soils, Bhattarai and co-authors recently identified denitrification as the major pathway [40].

The N_r_ measurements had to be carried out in the dark to avoid photochemical reactions. This has to be considered when interpreting the results. In other studies, it was shown that photosynthesis and respiration in illuminated cyanobacterial biocrusts recovered within minutes after water addition resulting in the formation of disparate oxygen microenvironments ranging from oxygen-supersaturated zones close to the surface to anoxic zones at 1–3 mm depth. Photosynthetic activity also affected the pH gradients in the biocrust. Local pH values up to 10.5 due to photosynthesis were measured, which may have consequences for the N_r_ emission patterns [93, 94].

The observed increase in transcript levels at T3 (Fig. 4c, d) might indicate that microorganisms remain in a state, enabling a rapid recovery when conditions become favourable for growth. Comparing transcript profiles of growing cells and substrate-deprived bacterial cells, it was observed that transcript levels of some genes, like *amoCAB* (*Nitrosomonas europaea*) or genes involved in metabolic pathways (*Mycobacterium tuberculosis*) did not change significantly under growth or starved conditions. Hence, the strategy to cope with periods of starvation appears to include maintenance of mRNA of key enzymes, like nitrification genes. This may allow a rapid recovery when substrate becomes available [56, 57, 95, 96]. A graphical representation of the temporal connection between microbial metabolic activity and the emissions of HONO and NO during desiccation is shown in Fig. S8.

The physicochemical and structural characteristics of the soil environment create microenvironments differing in soil hydration conditions, gas/liquid diffusion, and nutrient availability, causing a heterogeneous, patchy distribution of microbes in the soil [22, 97–102]. Moisture patchiness can result in aerobic and anaerobic microsites in close proximity, allowing different N-transformations to occur next to each other [103]. This patchy distribution of organisms and processes likely has a pronounced impact on rates and patterns of biogeochemical processes, like the emission of NO and HONO from biocrusts and soil, which needs to be investigated by studies on the microscale.

## Conclusions

Trace gas emission measurements over a desiccation cycle were linked to FGA, CARD-FISH, and soil analyses to obtain a deeper understanding of the biological processes involved in the observed N_r_ gas emissions. Our data illustrated that organisms involved in all major N-cycling processes became metabolically active within 30 min after wetting. The soil N content analyses showed a significant increase in NO_2_^−^-content, which likely served as a precursor for HONO and NO emissions peaking at relatively low water contents around 20% WHC. This is likely caused by a differential O_2_-dependent expression of nitrite as compared to nitrate reductase encoding genes during maximum HONO and NO emissions. Besides this, physiological responses in AOB (nitrification at atmospheric O_2_-levels, nitrifier denitrification under O_2_-limited conditions), and growth and transcriptional activity of AOA during the transition from O_2_-limited to oxic conditions, might contribute to the release of HONO and NO. Thus, our data suggest that AOA are major contributors to ammonia oxidation in biocrusts, and a differential expression of denitrification genes during drying causes an accumulation of NO_2_^−^, serving as a precursor for HONO and NO emissions.

## Supplementary information

Supplementary material 1

Supplementary material 2
