# Supplementary material for: Water-driven microbial nitrogen transformations in biological soil crusts causing atmospheric nitrous acid and nitric oxide emissions
Source: ISME J. 2021 Nov 11;16(4):1012–24. doi: 10.1038/s41396-021-01127-1 (PMC8941053; doi:10.1038/s41396-021-01127-1)
Supplement: Supplementary file 1 — Supplementary material 1 [file 41396_2021_1127_MOESM1_ESM.pdf]

Supplementary Material for manuscript

Water-driven microbial nitrogen transformations in biological soil crusts causing atmospheric nitrous acid and nitric oxide emissions

Maier, S.*[1,2], Kratz, A.M.*[2], Weber, J.[2], Prass, M. [2], Liu, F. [2,3], Clark, A.T. [1], Abed, R. [4], Su, H.[2], Cheng, Y.[2], Eickhorst, T.[5], Fiedler, S.[6] , Pöschl, U.[2], Weber, B. [1,2]

1 Institute of Biology, University of Graz, Graz, Austria

2 Multiphase Chemistry, Max Planck Institute for Chemistry, Mainz, Germany

3 School of Chemical & Biomolecular Engineering, Georgia Institute of Technology, Atlanta, Georgia 30332, United States

4 Sultan Qaboos University, College of Science, Biology Department, Al Khoud, Sultanate of Oman

5 Biology Department, University of Bremen, Bremen, Germany

6 Institute of Geography, Johannes Gutenberg University, Mainz, Germany

* contributed equally

This PDF file includes:

Materials and Methods……………………………….…………………………….. 2

Table S1 to S7………………………………………………………………………………………..….12

Figures S1 to S8……………………………………………………………….……………………...…… 20

Materials and Methods

Sampling

Cyanobacteria-dominated biocrusts were collected in small petri dishes (55 mm diameter and 15 mm height). For sampling, the bottom of the petri dish was placed upside down on the biocrust surface, pressed into the substrate, and with the help of a trowel pushed below, the biocrust part was lifted together with the underlying soil. In order to minimize metabolic activity, the samples were collected in an air-dried state and subsequently transported (darkened in a plastic box) by car and airplane to Germany. Before measurements, the samples were stored at the Max Planck Institute for Chemistry (MPIC, Mainz, Germany) for two and a half months (from the end of April to the beginning of July) at room temperature (25°C) in the dark.

Overall experimental setup

Dynamic chamber measurements were performed according to the study of Weber et al. [1] following the work of Su et al. [2] and Oswald et al. [3].

Nine measurements with three measurements/replicates for each stage of desiccation were carried out. The first sample set was removed from the chamber close to full water holding capacity (T1, early wetting; mean ~99% WHC; 20-30 minutes after wetting; Table S1), the second one at increasing emissions (at rising HONO and NO emissions before maximum was reached; T2, intermediate drying; mean ~30% WHC; 3.4 - 5 hours after wetting; Table S1), and the third one close to the end of desiccation cycle (T3, late drying; mean ~4% WHC; 6.3 - 8 hours after wetting; Table S1). Once the samples were removed from the chamber they were analyzed with different techniques (as described below) and therefore the whole material was used up for each measurement/replicate. Based on preliminary measurements, were full desiccation cycles were performed for the related samples (same biocrust type, study site and, collection date) and based on experiences, the second and third stage of desiccation (T2, T3) was estimated.

Dynamic chamber measurements

The dynamic chamber measurements are continuous measurements in the course of desiccation using a constant flow rate. During the experiments the chamber was kept in darkness in order to avoid photochemical reactions. The emissions and mixing ratios of NO, HONO, NO_2 (nitrogen dioxide), and H_2O were measured at the outlet of a Teflon (PFE) chamber (volume 0.047 m^3), which was purged with purified and dried air (PAG 003, Ecophysics, Duernten, Switzerland) at a flow rate of $1x10^{-4}$ m^3 s^{-1}.

In the course of desiccation, HONO was determined every 30 minutes, whereas NO was analyzed at 10-seconds interval. Fluxes F_N (ng m^{-2} s^{-1}) were calculated using the following formula [3]:

$$F_N = \frac{Q}{A} * \text{HONO [ppb]} * \frac{M_N}{Vm} \quad \text{or} \quad F_N = \frac{Q}{A} * \text{NO [ppb]} * \frac{M_N}{Vm}$$

F_N = flux of trace gas in terms of N (ng m^{-2} s^{-1})
Q = purging flow rate (m^3 s^{-1})
HONO/NO [ppb] = headspace mixing ratio
A = area of biocrust sample (m^2)
M_N = molar mass of nitrogen (g mol^{-1})
V_m = molar volume of air (m^3 mol^{-1})

For a time span of at least 20 minutes, before and after each measurement, zero air (empty chamber) was determined as a reference.
The water content of the samples was determined in the following way: In a first step, the total amount of water that could be held by the samples without dripping (= 100% WHC) was determined using a weight balance. Subsequently, the amount of H_2O (ppth) evapotranspirated from the sample was measured in 10-second intervals over the course of desiccation, using an infrared gas analyzer (LI-7000, LI-COR Biosciences GmbH, Germany). At the end of the measurement, the amount of water in the samples was checked gravimetrically to ensure that the calculations over the course of the measurements were correct (which was always the case).
Soil water contents and WHC were calculated using the following formulae:

$$SWC = \frac{m_{water} * 100\%}{m_{dry\ soil}} \qquad WHC = SWC \frac{m_{dry\ soil}}{m_{FC}}$$

SWC = soil water content [%]
WHC = water holding capacity [%]
m_{water} = mass of water in the sample [g]
$m_{dry\ soil}$ = mass of dry soil [g]
m_{FC} = mass of water at field capacity [g]. Field capacity is the amount of water held in a soil after gravitational water drainage stops.

In fully saturated samples, the mass of water in soil equals the field capacity [3].
NO and NO_2 were analyzed with a gas chemiluminescence detector, which was equipped with a blue light converter (Model 42C, Thermo Electron Corporation, USA; limit of detection $(LOD)_{NO} \approx 120$

ppt and LOD_{NO2} ≈ 300 ppt) [3]. HONO was detected spectrophotometrically using a long path absorption photometer (LOPAP, QUMA Elektronik & Analytik, Wuppertal, Germany; total accuracy 10% and detection limit ~3-6 ppt) [2]. The retention and response time accounted to 20 and 13 minutes, respectively. With this method, an acidic solution of sulphanilamide is used to sample HONO with a stripping coil directly connected to the chamber [1-6]. Upon reaction, HONO is immediately transformed into a diazonium salt, which serves as a precursor of a diazotization, which causes the formation of an azo dye. The concentration of the azo dye, which is equivalent to the concentration of HONO in the sampled air, is determined by means of VIS-spectrophotometry. A detailed description of the instrument was presented by Heland et al. [7] and Kleffmann et al. [8].

Microsensor measurements

Oxygen saturation was analyzed using oxygen microsensors (OX-100, Unisense A/S, Aarhus, Denmark) with a tip diameter of 100 µm. The measurement principle is based on diffusion of oxygen through a silicone membrane to an oxygen reducing cathode, which is polarized against an internal Ag/AgCl anode. An additional guard electrode ensures signal stability by removal of all oxygen diffusing towards the sensor from the internal electrolyte (Revsbech, 1989; Revsbech and Jørgensen, 1986).

We analyzed the oxygen saturation within the photoautotrophic layer (surface to 400 µm depth) and the heterotrophic layer (> 400 µm to 3000 µm) at T1 (~99% WHC) and T2 (~30% WHC). This was accomplished by vertical profiles at 200 µm steps. The water content (WHC) was determined with the help of a weight balance.

Nitrite and nitrate analyses

The NO_3^- and NO_2^- content of the biocrust samples was measured before (Pre) and after (Post) a desiccation cycle. In order to identify biological processes causing a potential accumulation of mineral N, also samples treated with methyl iodide (CH_3I) to suppress microbial activity were investigated. Methyl iodide treatment of samples was performed according to a procedure established for soil samples by Oswald et al. [3]. For this, biocrust samples were placed into a vacuum desiccator and 1 ml CH_3I (99% Reagent Plus, SIGMA-ALDRICH Chemie GmbH, Germany) was added in a separate beaker. Subsequently, a vacuum was generated and the samples were exposed to a high partial pressure of CH_3I for 24 hours [3]. NO_2^- and NO_3^- analyses of the soil were conducted according to DIN ISO 15923-1:2014-07 (DEV D49) and DIN 38405-9:2011-09 (DEV D9), respectively. Initially, each biocrust sample was ground with the help of a pestle and a mortar. Prior to the nutrient analysis, the dry matter content of each sample was determined. For analysis, 7 g of each sample were mixed with 28 ml of a 0.0125 mol/l $CaCl_2$ solution. In addition, a blank and two soil standard samples (7g internal lab reference material 13ME1 and 13ME4, respectively) were made. With the help of a shaker, all samples were mixed for 60 minutes, then they were filtered using nitrogen-poor filters,

which are low in possible nitrogen containing contaminants like NO_3^- and NO_2^- (Munktell Faltenfilter 292, Fa Ahlstrom-Munksjö, Helsinki, Finnland).

For the detection of NO_2^-, a NED (N-(1-Naphthyl)-ethylendiamine-dihydrochloride (Nr. 4342.1, Carl Roth, Karlsruhe, Germany) reagent was prepared. For this, 25 ml phosphoric acid (9179.1, Carl Roth, Karlsruhe, Germany) were mixed with 2.5 g sulphanilamide (Nr. 4716.1, Carl Roth, Karlsruhe, Germany) and 0,125 g NED and solved in 250 ml ultrapure water (18.2 MΩ) in a 500 ml volumetric flask. Subsequently the solution was filled up to 500 ml with ultrapure water. Standard solutions of NO_2^- (100µl of NO_2^- standard solution and the 0.0125 mol/l $CaCl_2$ solution were diluted to 100 ml in a volumetric flask) in the concentrations 0, 0.1, 0.2, 0.4, 0.6, 0.8, 1.0 mg/l were prepared. 1 ml of the NED reagent was added to each sample, standard soil, blank, and the standards. The samples were mixed with the help of a vortexer and after 10 minutes of reaction time, the extinction values (wavelength: 540 nm) of all samples and standards were determined using a photometer (Photometer Specord50, Analytik Jena AG, Jena, Germany).

Similar to the nitrite analysis, a NO_3^- standard solution, a DMP reagent (0,06g DMP diluted in 50ml acetic acid; Nr. 3738.5, Carl Roth, Karlsruhe, Germany), an acid mixture (phosphoric and sulfuric acid at the ratio 1:1; Nr. 9179.1 and 9316.1, respectively, Carl Roth, Karlsruhe, Germany) were prepared. Subsequently a standard series of seven standards was prepared (0, 10, 20, 40, 60, 80, 100 mg/ l). 8 ml of acid mixture were added to samples, standard soil, and blanks (1 ml), plus standard series and swayed but not shaken. Afterwards, 1 ml of the DMP reagent was added to all samples, swayed, and incubated at room temperature for 10 minutes. The extinction values of all samples and standards were determined at 338 nm with a photometer (Photometer Specord50).

The conversion of mg/l into mg/kg is described in the following:

mg/kg NO_2^- = mg/l NO_2^- x volume of extraction solution / soil mass

mg/kg NO_3^- = mg/l NO_3^- x volume of extraction solution / soil mass

The conversion factor from nitrite to nitrite nitrogen (NO_2-N) and nitrate to nitrate nitrogen (NO_3-N) amounts to 0.3045 and 0.2259, respectively.

Detection and quantification of bacterial and archaeal populations in soil samples by catalyzed reporter deposition-fluorescence *in situ* hybridization (CARD-FISH)

Fixation procedure: Preparatory steps for CARD-FISH were performed based on a procedure for soil samples established by Eickhorst and Tippkötter [9] and Schmidt et al. [10]. Three replicate samples were taken at T1 and T2 and of each sample three technical replicates were prepared. All samples were divided into two biocrusts layers (upper layer; 0 - ~5 mm depth: photoautotrophic biocrust section; lower layer ; ~5 – 10 mm depth: heterotrophic biocrust section). 500 mg of soil of each layer were transferred into 2 mL plastic micro test tubes (Eppendorf, Hamburg, Deutschland). For fixation, the samples were incubated in a 4% formaldehyde solution in 1 x PBS buffer at 4°C for 2.5 h. Subsequently, the samples were centrifuged at 10,000 x g and 4°C for 5 min, and were then washed

two times in 1 x PBS buffer (1.5 mL), with each step being followed by a centrifugation with the same characteristics. Afterwards, the samples were stored in 1.5 mL of 1 x PBS/ethanol (1:1; v:v) solution at -20°C until further analysis.

Preparation of soil samples on filter membranes: The soil samples were defrosted at RT (~ 5 minutes) and a 1:10 dilution in 1 x PBS/EtOH (v/v) was then subjected to ultrasound twice for 30 seconds with a 30 second break in-between. Then, 50 µL of the sample were diluted in 10 mL H_2O_{MQ} and filtered onto a polycarbonate filter (Isopore Membrane, ø 25 mm, 0.2 µm pore size, Merck, Darmstadt, Germany) applying a vacuum of 800 mbar. The air-dried filters were briefly immersed into 0.2% low melting point agarose (SIGMA-ALDRICH, Darmstadt, Germany) and dried at room temperature [9-11].

Permeabilization of cell walls: The filters were first incubated in a lysozyme solution [10 mg mL^{-1} lysozyme (SIGMA-ALDRICH, Darmstadt, Germany), 50 mM EDTA, 100 mM Tris-HCl, H_2O_{MQ}] for 60 min and in an achromopeptidase solution [60 U mL^{-1} achromopeptidase (SIGMA-ALDRICH, Darmstadt, Germany), 10 mM NaCl, 10 mM Tris-HCl, H_2O_{MQ}] at 37°C for 30 min and finally washed in H_2O_{MQ} [9-11].

Inactivation of endogenous peroxidases: Filters were incubated in methanol containing 0.15% (v/v) H_2O_2 for 30 min at RT. The filters were immersed in H_2O_{MQ} for 5 min, and then dipped into 98% EtOH and allowed to dry. After this step, filters were cut into 12 pieces and were stored at -20 °C until *in situ* hybridization and quantification of cells [9, 10].

In situ hybridization: A hybridization buffer set to a probe specific formamide concentration (see Table S2) [0.9M NaCl; 20 mM Tris–HCl, pH 8.0; 100 mg mL^{-1} dextran sulphate (Merck, Darmstadt, Germany); 1% (w/v) blocking reagent (SIGMA-ALDRICH, Darmstadt, Germany); 0.01% (v/v) sodium dodecyl sulphate; 35%/50% (v/v) formamide; H_2O_{MQ}] was prepared. The stock solution of the blocking reagent (10% w/v) was prepared in maleic acid buffer (100 mM maleic acid, 150 mM NaCl, pH 7.5). The hybridization buffer (400 µL) was mixed with 1.5 µL of the probe working solution (50 ng μL^{-1} horseradish peroxidase (HRP)-labeled oligonucleotide probes obtained from Biomers, Ulm, Germany) and added to the filter sections in 0.5 mL reaction tubes. Hybridization was done at 35°C for 2 h on a tube rotor in an incubator. Subsequently, filter sections were incubated in the prewarmed washing buffer (5 mM EDTA [pH 8.0], 20mM Tris–HCl [pH 8.0], 0.01% (v/v) SDS, a probe-dependent amount of 5 M NaCl (see Table S2), filled up to 50 mL with H_2O_{MQ}) at 37°C for 10 min, washed in H_2O_{MQ} (RT) and then transferred into 0.05% (v/v) Triton X- PBS buffer [25 µL Triton-X100 (Fisher Scientific, Vienna, Austria), 50 mL 1 x PBS] for 5 min at RT [9-11]. Details on HRP-labeled oligonucleotide probes and hybridization conditions are given in Table S2. As the utilized

probes have been previously described and registered in probeBase, a specificity test was not performed.

Tyramide signal amplification: The filter sections were incubated in 500 µL amplification buffer [100 mg mL^{-1} dextran sulfate, 0.1% (w/v) blocking reagent, 2 M NaCl, 1x PBS], containing 0.0015% (v/v) H_2O_2 and 2 µL mL^{-1} of Alexa Fluor 488–labeled tyramide solution (Thermo Fisher Scientific, Massachusetts, US), at 37°C for 20 min while rotating. Subsequently, the filter sections were dipped in 0.05% (v/v) Triton X-PBS for 5 min, washed in H_2O_{MQ} for 5 min, and dehydrated with 98% EtOH. Working steps with the light-sensitive tyramide solutions were carried out under a fume hood in a darkened room [9-11].

Counterstaining with DAPI and Fluorescence microscopy: Dry filter sections were placed on glass slides, Vectashield (VECTASHIELD Antifade Mounting Medium with DAPI, VECTOR laboratories) was added and then filter sections were covered with cover slips. The samples were analyzed using a Nikon Ti2-E inverse research microscope (Nikon, Tokyo, Japan) equipped with a LED light source (Lumencor Sola SE II 365HBO, Nikon) and an APO 60x Lambda oil objective (Nikon). For image recording, a camera (DS-Qi2, Nikon) connected to an imaging software (NIS-Elements, Nikon) was used. Signals of AlexaFluor 488-labeled tyramides were detected with a F46-018NXL EGEP ET longpass filter set (470/ 500 LP nm ex/em). UV excitation and a F36-499NXL DAPI HC longpass filter set were used for visualization of the cells.

Quantification: An eyepiece reticle (net micrometer covering an area of 10 x 10 mm, Nikon, Tokyo, Japan) was used for cell counting. For 10 microscopic fields of sight, that were randomly chosen on the filter sections, the cells in 100 squares were counted (10 areas of 10 x 10 mm; 1000 squares in total/filter section). Taking into account the dilution of the soil samples in the course of the fixation, sonication and filtration procedure, these cell numbers were extrapolated to cells per gram of soil (dry weight).

Statistics: To test for significant differences in cell counts among measurements, we applied a Bayesian hierarchical model, fitted via the Stan Hamiltonian Monte Carlo sampler [12], implemented using the “brms” package (version 2.13.5; [13]) in the R programming language (version 4.0.2; [14]). We fit separate models for EUB, Archaea, and NOB, with soil layer and stop time coded as categorical variables. Differences among treatments were quantified based on the posterior distribution of contrasts between these categorical variables, e.g. as the difference between estimated cell counts for UL (T1) vs. UL (T2). Pairs of treatment for which this difference did not include zero within the one-tailed 95% credibility interval were interpreted as being “significanty” different from one another. For all models, we ran 4 chains for 2000 iterations, and discarded the first 1000 iterations as a burn-in

period. To account for pseudoreplication, we included a "random intercept term" (i.e. fitted categorical variables constrained by a shared Gaussian prior) for sample ID nested within dish ID, of the form ~(1|Dish/Sample). All other priors were left as the default (i.e. improper flat priors). Visual inspection revealed no convergence problems across models, and for all parameter estimates, we found high effective sample sizes (2087 – 3568) and Rhat < 1.01, indicating excellent convergence. No divergent transitions or other errors were reported by the optimiser. The R script is available on Zenodo (https://zenodo.org/record/5110117#.YPGqpOgzbb0).

GeoChip functional gene microarray

The functional gene microarray (FGA) GeoChip allows to study the genes involved in the biogeochemical cycling of nitrogen, carbon, sulfur, and phosphorus [15, 16]. The GeoChip 5.0 version, manufactured by Agilent Technologies (Santa Clara, CA, USA), has 57,000 oligonucleotide probes (60K). Only genes for proteins containing catalytic subunits or active sites are included [16]. The gene markers used for the detection of nitrification included *amoA* and *hao*, whereas *narG*, *nirK*, *nirS*, *norB* and *nosZ* indicated denitrification processes.

RNA extraction, cDNA synthesis, labeling

Before extraction, the samples were placed on liquid N_2 and homogenized using a RNase/DNase free polystyrene spatula. From 2 g of soil, taken from several areas and over the entire height (15 mm) of the petri dish, RNA was extracted using the RNeasy PowerSoil® Total RNA kit (Qiagen, Venlo, Netherlands), following manufacturer´s instructions. We also included a negative control (without soil).

The purity and concentration of the extracted RNA was determined using the Multiskan GO UV/VIS spectrophotometer (Thermo Fisher Scientific, Waltham, MA, US) by measuring the absorbance at 260 and 280 nm as well as with the Qubit RNA HS Assay Kit using the Qubit® 2.0 Fluorometer (Thermo Fisher Scientific, Waltham, MA, US). RNA integrity was checked with agarose gel electrophoresis.

The GeneJET RNA Cleanup and Concentration Micro Kit (Thermo Fisher Scientific, Waltham, MA, USA) was used to concentrate RNA samples. Residual DNA was digested by treating the sample with RNase-free DNaseI (AMPD1, Sigma-Aldrich, Darmstadt, Germany). RNA reverse transcription was performed using the Maxima H Minus Double-Stranded cDNA Synthesis kit (Thermo Fisher Scientific, Waltham, MA, USA). We performed control reactions that did not contain reverse transcriptase enzyme to confirm there was no DNA contamination in the cDNA preparations. When performing PCR, targeting the 16S rRNA gene, we used HPLC purified, universal primers 515f (5´-GTG YCA GCM GCC GCG GTAA 3´) and 806r (5´- GGA CTA CNV GGG TWT CTA AT -3´) targeting the hypervariable region V4 from Bacteria and Archaea (Sigma-Aldrich, Germany). A PCR mix of 25 µl was prepared containing 1 x Fast Start High Fidelity Buffer, 1.25 U High Fidelity Enzyme, 200 µM dNTPs, 0.4 µM primers (Roche Diagnostics, Germany), and PCR-grade water (Jena BioScience, Germany). Thermal cycling conditions were for initial denaturation at 95°C for 3 min

followed by 30 cycles for denaturation at 95°C for 45s, annealing at 55°C for 45s, and extension at 72°C for 1 min with a final extension of 7 min at 72°C. No PCR amplicons could be obtained from samples when reverse transcriptase was omitted from the reactions.

cDNA extracts were stored at -20˚C until use. cDNA was used for functional gene array hybridization. Sample labeling, hybridization, scanning, and image processing were performed at the company Glomics Inc. (Norman, OK, USA). Labeling of DNA for microarray hybridization was performed with fluorescent cyanine dyes [16].

Data Processing and Analysis

Prior to data analysis, normalization was performed to take into account unequal quantities of starting RNA, differences in labeling or detection efficiencies between the fluorescent dyes, and systematic differences across datasets [17]. For microarray data normalization and comparison of gene array data across different samples and stages during desiccation, the common oligonucleotide reference standard (CORS) method was applied [16, 18]. The CORS is an artificial sequence probe, which is co-spotted with each gene probe. The complementary CORS target is labeled with a fluorescent dye different from that of the sample and is spiked into each sample before hybridization [16, 18]. For data normalization, the average signal intensity of CORS was calculated for each array and the maximum average value was used to normalize the signal intensity of samples in each array. Secondly, for each array the sum of the signal intensity of samples was calculated. The maximum sum value was applied to normalize the signal intensity of all spots in an array, generating a normalized value for each spot. Spots were considered as positive if the signal-to-noise ratio was ≥2.0 [SNR = (signal mean – background mean)/background standard deviation], and the signal intensity was at least 1.3 times the background. Furthermore, spots with signal intensities less than ~200 were removed [16]. Of 24 983 positive spots, 5 275 (21.1%) were excluded because they were detected in only one of the three replicates. In the current study, only probes involved in the biogeochemical cycling of nitrogen have been analyzed. The Geochip data analysis was performed with a software pipeline developed by the company Glomics. The pipeline includes tools to perform descriptive statistics, such as relative abundances of genes/gene categories/subcategories, and richness, alpha, and beta diversity indices of functional genes [15, 16]. A logarithmic transformation was carried out to improve the characteristics of the data distribution and variables were scaled before measuring inter-observation dissimilarities.

Class comparison/differential expression: The microarray experiment was designed as class comparison experiment [19, 20]. We looked for genes with statistically significant differences between the three stages during desiccation. For differential expression analysis, a global test, linear models for microarray (limma method), was applied, which analyzes each probe separately [19, 20]. To assess the differential expression of each probe by means of limma, the moderated t- statistic was applied, where the standard errors have been moderated across genes using a Bayesian model [21, 22]. As testing thousands of genes is likely to produce hundreds of false positives, multiple testing correction is necessary. The chosen approach was to control the false discovery rate (FDR; e.g. Benjamini-

Hochberg; BH(p) = significant if ≤ α). The FDR is a less restrictive approach than the family wise error rate procedure [19].

Clustering: Clustering is a method used to identify spatial or temporal expression patterns or identify co-regulated genes. Unsupervised clustering techniques include hierarchical clustering, self-organizing maps (SOM), and k-means. Hierarchical clustering can be used to find a partition of the samples as well as to find groups of co-regulated genes [19]. The classification of observations into groups requires calculation of the distance or (dis)similarity between each pair of observations. The distance measures Euclidean and Manhattan distance as well as correlation-based distances, including Pearson and Spearman correlation distance, were computed using dist() R base function (Kassambara 2017, R stats package) and visualized using the R fviz_dist function (R factoextra package). The hierarchical cluster analysis (HCA) approach was used to group samples based on their similarity. Distance matrices generated by the function dist() were used to produce hierarchical trees using the R base function hclust(). As agglomeration (linkage) method, complete linkage was used [23]. For visualization of hierarchical clustering, a dendrogram was computed using fviz_dend() in factoextra R package. To assess whether the cluster tree reflects the data, we computed the correlation between the cophenetic distances and the distance data calculated with the dist() function [23]. Internal (Silhouette coefficient) and external cluster validation statistics were calculated (corrected Rand index). The silhouette coefficient (S_i) ranges from 1 to -1 and measures how similar a sample *i* is to the other samples in its clusters. A value close to 1 indicates that the sample *i* is similar to the other objects in its group. The corrected Rand index varies from –1, indicating no agreement to 1, indicating perfect agreement between clusters and an external reference (sample design, three replicates per stage during desiccation) [23].

Mass spectrometry-based metaproteomics

For the extraction of extracellular and intracellular microbial protein, the NoviPureTM Soil Protein Extraction Kit (Qiagen, Netherlands) was used. Proteins were extracted from 5 g of soil and dry pellets were stored at -20°C. The dried residues were resuspended in 500 µL ammonium bicarbonate buffer (25 mM) and subjected to size exclusion chromatography (PD MiniTrapTM G-25, exclusion limit 5 kDa, GE Healthcare, Germany) according to the supplier´s instruction (gravity protocol). For the measurement of the protein concentration, a bicinchoninic acid (BCA) assay (PierceTM BCA Protein Assay Kit, Thermo Scientific) was used. The assay was performed in 96-well microplates. 10 µL of sample were used and incubated at 37°C for 30 min. The absorbance was measured at 560 nm using a microplate photometer (Thermo Scientific Multiskan EX). Lyophilisation was performed overnight (Christ Alphaa 2-4 LD, Germany). Subsequently, in-solution digestion with trypsin was performed overnight. Trypsin stock solution (1 µg/µL) was added at 1:50 enzyme:substrate and incubated for 18 h at 37°C. The extracts were desalted with Pierce® C18 spin columns (Thermo scientific) according to the manufacturer´s instructions.

Peptides were separated by nanoflow liquid chromatography on an EASY-nLC 1000 system (Thermo) coupled to a Q Exactive Plus mass spectrometer (Thermo) [24] at the proteomics core facility of the institute of Molecular Biology (IMB), Mainz. Separation was achieved by a 25-cm capillary (New Objective) packed in-house with ReproSil-Pur C18-AQ 1.9-µm resin (Dr. Maisch). Peptides were separated chromatographically by a 105 min gradient from 2% to 40% acetonitrile in 0.5% formic acid with a flow rate of 250 nl/min. Spray voltage was set between 2.4 and 2.6 kV. The instrument was operated in the data-dependent mode (DDA) performing a top10 MS/MS per MS full scan. Isotope patterns with unassigned and charge state 1 were excluded. MS scans were conducted with 70,000 and MS/MS scans with 17,500 resolution.
Data were analyzed using MaxQuant (version 1.5.2.8) with default settings [25]. Carbamidomethyl was defined as fixed modification, acetylation and oxidation where defined as variable modification. The search was performed against the Swiss-Prot database, which contains reviewed and manually annotated protein sequences, downloaded in April 2017 (~590000 sequences). MaxQuant results where further filtered my removing all reverse database binders, known contaminants and proteins only identified by site modification. As an additional filtering step we removed all protein groups detected with less than 2 peptides (minimum 1 unique).
Each protein entry is linked to secondary databases with functional information including the Gene Ontology (GO) database, which describes gene products by using three categories: biological processes, cellular components, and molecular function.

Table S1 HONO and NO emissions: **a**: Sample information wetting and drying experiment (SOD = Stage of desiccation; SWC = Soil water content; WHC = Water holding capacity; Stop = End of measurement; Max = Maximum values; Int = Integrated values); **b**: Statistical results for HONO and NO emissions (Data transformation of NO_{Max} with sin(x)); **c**: Statistical results for nutrient values (Data transformation with │ln(x)│; NO_3^--N: F = 3.448, DF = 3, p = 0.071; NO_2^--N:, F = 12.633, DF = 3, p = 0.002; methyl iodide (CH_3I) treated samples = treated).

a

**SOD + #Nr.**	**Water [g] Start**	**Water [g] Stop**	**SWC [%] Start**	**SWC [%] Stop**	**WHC [%] Stop**	**NO Stop [ng N m^{-2} s^{-1}]**	**HONO Stop [ng N m^{-2} s^{-1}]**	**NO_{Max} [ng N m^{-2} s^{-1}]**	**$HONO_{Max}$ [ng N m^{-2} s^{-1}]**	**NO_{Int} [ng N m^{-2} s^{-1}]**	**$HONO_{Int}$ [ng N m^{-2} s^{-1}]**
1 #1	16.29	16.07			98.6						
1 #2	20.52	20.30			98.9						
1 #2	18.98	18.77			98.9						
2 #1	29.16	4.99	36.3	6.2	17.1	76.4	37.8	76.4	37.8	10348.0	5384.4
2 #2	27.44	8.94	36.6	11.9	32.6	59.1	25.4	59.1	25.4	7033.0	3297.9
2 #3	28.08	10.91	35.7	13.9	38.9	59.1	23.5	59.1	23.5	3941.9	1702.5
3 #1	20.63	0.96	33.6	1.6	4.7	26.6	18.5	136.7	74.4	35681.0	22616.8
3 #2	19.19	0.54	31.1	0.9	2.8	49.4	23.1	186.5	84.4	50891.7	23774.9
3 #3	27.23	0.87	32.1	1.0	3.2	16.0	12.6	137.2	56.7	51515.2	23305.7

b

	***t*-statistics**	**DF**	***p* value**
$HONO_{Max}$: T2 vs. T3	-4.64005	4	0.00973
NO_{Max}: T2 vs. T3	-2.82314	4	0.04768
$HONO_{Int}$: T2 vs. T3	-17.68727	4	$6.00219e^{-5}$
NO_{Int}: T2 vs. T3	-7.07957	4	0.0021

c

**NO_3^--N**	Pre control	Post control	Pre treated	Post treated
Pre control		0.1105	0.99991	0.99941
Post control			0.10151	0.12971
Pre treated				0.99793
Post treated				
**NO_2^--N**	Pre control	Post control	Pre treated	Post treated
Pre control		**0.00694**	0.68278	0.55071
Post control			**0.00181**	**0.04329**
Pre treated				0.13494
Post treated				

Table S2 Horseradish peroxidase-labeled oligonucleotide probes and hybridization conditions

Target group	Probe name	Sequence (5´-3´)	References	FA (%)	NaCl (mM)
**Most bacteria**	EUB338	GCTGCCTCCCGTAGGAGT	Amann et al. [26]	35	42
**Planctomycetales**	EUB338 II	GCAGCCACCCGTAGGTGT	Daims et al. [27]	35	42
**Verrucomicrobiales**	EUB338 III	GCTGCCACCCGTAGGTGT	Daims et al. [27]	35	42
**Archaea**	Arch915	GTGCTCCCCCGCCAATTCCT	Burggraf et al. [28]	35	42
**Nitrite-oxidizing bacteria (NOB), Phylum Nitrospirae competitor+**	Ntspa712	CGCCTTCGCCACCGGTGTTCC	Daims et al. [29]	50	9
**Nitrite-oxidizing bacteria (NOB), Phylum Nitrospirae**	Ntspa712	CGCCTTCGCCACCGGCCTTCC	Daims et al. [30]	50	9
**AOB**	Nso1225	CGCCATTGTATTACGTGTGA	Mobarry et al. [31]	35	42
**None (control probe)**	NONEUB	ACTCCTACGGGAGGCAGC	Wallner et al. [32]	35	42

FA: concentration of formamide in the hybridization buffer; NaCl: concentration of NaCl in the washing buffer

+Unlabelled competitor oligos were used, which suppress unspecific probe binding to non-target sequences.

Table S3 Number of probes detected on the GeoChip functional microarray.

	Timepoint 1			Timepoint 2			Timepoint 3		
	T1_1	*T1_2*	*T1_3*	*T2_1*	*T2_2*	*T2_3*	*T3_1*	*T3_2*	*T3_3*
**No. probes detected**	14662	14672	12692	18427	14654	14755	17094	18363	17940
**%**	25.7	25.7	22.3	32.3	25.7	25.9	30.0	32.2	31.5
**After filtering:**									
**No. probes detected**	12380	12595	11916	15484	13914	13970	16035	16845	16680
**%**	21.7	22.1	20.9	27.2	24.4	24.5	28.1	29.6	29.3
**No. probes detected (gene category Nitrogen)**	1590	1632	1561	2013	1819	1817	2089	2208	2162
**%**	2.8	2.9	2.7	3.5	3.2	3.2	3.7	3.9	3.8
**% probes (gene category Nitrogen) of detected probes**	12.8	13.0	13.1	13.0	13.1	13.0	13.0	13.1	13.0

Table S4 Number of detected overlapping probes between samples

	Timepoint 1			Timepoint 2			Timepoint 3		
**Sample name**	*T1_1*	*T1_2*	*T1_3*	*T2_1*	*T2_2*	*T2_3*	*T3_1*	*T3_2*	*T3_3*
T1_1		11078 (80.9%)	10411 (76.0%)	11257 (68.9%)	10442 (66.8%)	10653 (68.8%)	11422 (68.2%)	11369 (64.7%)	11521 (66.7%)
T1_2			10616 (77.5%)	11533 (70.8%)	10739 (69.1%)	10878 (70.4%)	11687 (70.1%)	11735 (67.4%)	11688 (67.5%)
T1_3				11078 (68.9%)	10377 (68.2%)	10544 (69.7%)	11113 (67.0%)	11000 (62.9%)	11135 (64.8%)
T2_1					13469 (86.12%)	13533 (86.5%)	13796 (79.3%)	13952 (77.3%)	13981 (78.3%)
T2_2						11991 (76.7%)	12539 (73.2%)	12540 (70.0%)	12597 (71.2%)
T2_3							12825 (75.9%)	12886 (73.1%)	12982 (74.7%)
T3_1								14918 (84.6%)	14754 (83.7%)
T3_2									15549 (88.2%)
T3_3									

Table S5 Number of detected overlapping probes between samples. Only probes involved in the biogeochemical cycling of nitrogen were considered.

	Timepoint 1			Timepoint 2			Timepoint 3		
**Sample name**	*T1_1*	*T1_2*	*T1_3*	*T2_1*	*T2_2*	*T2_3*	*T3_1*	*T3_2*	*T3_3*
T1_1		1412 (78.4%)	1343 (74.6%)	1451 (67.7%)	1353 (66.1%)	1369 (67.5%)	1468 (66.7%)	1473 (63.6%)	1475 (65.1%)
T1_2			1382 (76.7%)	1496 (70.0%)	1405 (69.0%)	1405 (69.1%)	1512 (68.9%)	1522 (66.0%)	1499 (65.7%)
T1_3				1446 (68.3%)	1368 (68.3%)	1384 (69.8%)	1455 (66.7%)	1445 (62.5%)	1444 (63.7%)
T2_1					1770 (86.3%)	1767 (86.2%)	1815 (79.9%)	1835 (77.3%)	1821 (77.8%)
T2_2						1575 (76.8%)	1658 (74.1%)	1664 (70.8%)	1648 (71.0%)
T2_3							1677 (75.7%)	1686 (72.5%)	1687 (74.0%)
T3_1								1959 (84.3%)	1912 (82.3%)
T3_2									2033 (87.5%)
T3_3									

Table S6 Number of taxonomic ranks (mean ± SD). Different lowercase letters indicate statistically significant differences among time points.

Timepoint	No. superkingdoms	No. phyla	No. classes	No. orders	No. families	No. genera	No. species
**T1**	3	25.3±0.5	48.3±1.3 a	87.0±1.6 a	138.0±3.7 a	277.3±6.1 a	320.7±8.2 a
**T2**	3	25.7±1.3	49.3±1.3 a	91.0±2. 5 ab	157.0±4.2 b	309.3±10.4 b	366.7±15.8 b
**T3**	3	26.7±0.5	53.3±0.5 b	96.0±0.8 b	165. 7±1.9 b	337.0±0.8 c	410.0±4. 6 c

Table S7 N-transforming microorganisms detected in biocrusts at different stages during desiccation (T1, T2, T3, *n* =3) using a functional gene microarray. At T2 the samples had a mean WHC of ~ 30% and at T3 ~ 4%.

Phylum	Family	T1 (1)	T1 (2)	T1 (3)	T2 (1)	T2 (2)	T2 (3)	T3 (1)	T3 (2)	T3 (3)
Crenarchaeota	*Desulfurococcaceae*	1	0	1	1	0	1	1	1	1
Crenarchaeota	*Sulfolobaceae*	0	1	1	1	1	1	1	1	0
Euryarchaeota	*Archaeoglobaceae*	0	0	0	1	1	1	1	1	1
Euryarchaeota	*Halobacteriaceae*	11	14	13	17	15	13	20	18	19
Euryarchaeota	*Methanococcaceae*	1	1	0	1	1	0	1	1	1
Euryarchaeota	*Methanosarcinaceae*	3	2	2	3	3	2	4	3	2
Euryarchaeota	*Methanocellaceae*	2	2	0	3	2	3	3	3	3
Euryarchaeota	*Methanosaetaceae*	1	1	1	1	1	1	1	1	1
Euryarchaeota	*Methanomicrobiaceae*	0	0	0	0	0	0	1	1	1
Euryarchaeota	*Methanoregulaceae*	0	0	0	1	1	1	1	0	1
Euryarchaeota	*Methanopyraceae*	0	1	1	1	1	1	1	1	1
Euryarchaeota	*Thermococcaceae*	3	3	3	3	3	3	3	3	3
Thaumarchaeota	*Cenarchaeaceae*	1	1	1	1	1	1	1	0	1
Acidobacteria	*Acidobacteriaceae*	1	1	1	1	0	1	1	1	1
Actinobacteria	*Gordoniaceae*	15	12	13	16	15	16	14	15	15
Actinobacteria	*Corynebacteriaceae*	13	16	11	16	16	12	16	15	15
Actinobacteria	*Streptomycetaceae*	12	11	11	13	10	13	13	13	12
Actinobacteria	*Mycobacteriaceae*	11	9	10	11	9	12	12	12	12
Actinobacteria	*Nocardiaceae*	7	7	7	10	9	8	9	10	10
Actinobacteria	*Pseudonocardiaceae*	7	8	8	8	8	8	8	8	8
Actinobacteria	*Micrococcaceae*	5	6	6	7	7	6	8	8	8
Actinobacteria	*Actinomycetaceae*	3	3	2	4	3	3	5	5	4
Actinobacteria	*Frankiaceae*	3	4	4	5	4	5	5	5	5
Actinobacteria	*Micromonosporaceae*	2	2	1	2	1	2	2	2	2
Actinobacteria	*Propionibacteriaceae*	2	2	2	2	1	2	3	2	3
Actinobacteria	*Dermacoccaceae*	1	1	1	1	1	1	1	1	1
Actinobacteria	*Dermatophilaceae*	1	1	0	1	1	1	1	1	1
Actinobacteria	*Intrasporangiaceae*	1	1	1	1	1	1	1	1	1
Actinobacteria	*Microbacteriaceae*	1	1	1	1	1	1	1	1	1
Actinobacteria	*Thermomonosporaceae*	1	1	0	2	2	1	2	2	2
Actinobacteria	*Coriobacteriaceae*	1	1	1	3	2	3	2	2	2
Actinobacteria	*Dermabacteraceae*	0	1	1	1	1	1	1	1	1
Actinobacteria	*Geodermatophilaceae*	0	0	0	1	1	1	0	0	0
Actinobacteria	*Nocardioidaceae*	0	0	0	1	1	1	1	1	0
Aquificae	*Aquificaceae*	1	1	0	1	1	1	1	1	1
Bacteroidetes	*Prevotellaceae*	3	5	5	7	6	7	9	9	8
Bacteroidetes	*Bacteroidaceae*	2	2	2	3	3	2	2	3	3
Bacteroidetes	*Porphyromonadaceae*	2	3	2	2	2	2	3	3	3
Bacteroidetes	*Rikenellaceae*	2	2	2	2	2	2	2	3	3
Bacteroidetes	*Cytophagaceae*	3	2	3	3	2	3	4	4	3
Bacteroidetes	*Cyclobacteriaceae*	1	1	1	1	1	1	1	1	0
Bacteroidetes	*Flavobacteriaceae*	6	3	5	5	6	5	6	8	8
Bacteroidetes	*Flavobacteriaceae*	6	3	5	5	6	5	6	8	8
Bacteroidetes	*Blattabacteriaceae*	0	0	0	1	1	1	0	0	0
Bacteroidetes	*Cryomorphaceae*	0	0	0	0	0	0	1	1	1

Table S7 (cont.)

Phylum	Family	T1 (1)	T1 (2)	T1 (3)	T2 (1)	T2 (2)	T2 (3)	T3 (1)	T3 (2)	T3 (3)
Bacteroidetes	*Sphingobacteriaceae*	3	3	2	2	2	2	2	2	2
Bacteroidetes	*Sphingobacteriaceae*	3	3	2	2	2	2	2	2	2
Bacteroidetes	*Rhodothermaceae*	0	0	0	0	0	0	1	1	1
Chlamydiae	*Waddliaceae*	0	1	1	0	0	0	1	1	1
Chlorobi	*Chlorobiaceae*	1	2	2	2	1	2	3	2	3
Chloroflexi	*Caldilineaceae*	1	1	2	2	2	2	2	2	2
Chloroflexi	*Chloroflexaceae*	1	0	1	0	0	0	1	1	1
Chloroflexi	*Sphaerobacteraceae*	1	1	0	1	0	1	1	1	1
Chloroflexi	*Thermomicrobiaceae*	1	1	1	1	1	1	2	1	2
Chrysiogenetes	*Chrysiogenaceae*	1	1	1	1	1	1	1	1	1
Cyanobacteria	*Nostocaceae*	4	4	3	7	5	4	6	7	6
Cyanobacteria	*Prochlorococcaceae*	2	2	2	3	2	2	3	2	3
Cyanobacteria	*Scytonemataceae*	1	1	1	1	1	1	1	1	1
Deinococcus-Thermus	*Deinococcaceae*	3	3	2	3	2	3	3	3	3
Deinococcus-Thermus	*Thermaceae*	0	0	0	2	2	1	1	3	2
Fibrobacteres	*Fibrobacteraceae*	1	1	1	1	1	1	1	1	1
Firmicutes	*Paenibacillaceae*	8	7	6	11	9	10	10	11	10
Firmicutes	*Bacillaceae*	7	10	9	9	9	8	11	11	12
Firmicutes	*Lactobacillaceae*	3	4	3	4	4	2	4	5	5
Firmicutes	*Streptococcaceae*	2	2	0	2	2	0	1	2	1
Firmicutes	*Staphylococcaceae*	1	0	1	1	1	1	2	1	2
Firmicutes	*Thermoactinomycetaceae*	1	0	1	1	1	0	1	1	0
Firmicutes	*Alicyclobacillaceae*	0	0	0	2	2	1	2	1	2
Firmicutes	*Planococcaceae*	0	0	0	1	1	1	0	0	0
Firmicutes	*Aerococcaceae*	0	0	0	1	1	0	1	1	1
Firmicutes	*Peptococcaceae*	10	8	7	11	10	11	11	12	11
Firmicutes	*Clostridiaceae*	5	7	6	4	6	5	5	5	6
Firmicutes	*Heliobacteriaceae*	3	3	3	3	2	2	3	3	3
Firmicutes	*Syntrophomonadaceae*	1	1	0	1	1	1	2	2	2
Firmicutes	*Clostridiales Family XI. Incertae Sedis*	0	0	0	1	1	1	3	1	2
Firmicutes	*Clostridiales Family XVIII. Incertae Sedis*	0	0	0	0	0	0	0	0	0
Firmicutes	*Eubacteriaceae*	0	0	0	1	0	1	1	1	1
Firmicutes	*Lachnospiraceae*	0	0	0	0	0	0	1	1	0
Firmicutes	*Ruminococcaceae*	0	0	0	1	0	1	2	3	3
Firmicutes	*Veillonellaceae*	6	6	5	8	6	8	10	10	10
Gemmatimonadetes	*Gemmatimonadaceae*	1	1	1	1	0	1	1	1	1
Ignavibacteriae	*Melioribacteraceae*	0	0	0	1	1	0	0	0	0
Lentisphaerae	*Lentisphaeraceae*	0	0	0	1	1	1	1	1	1
Nitrospirae	*Nitrospiraceae*	3	3	2	2	3	3	2	2	2
Planctomycetes	*Phycisphaeraceae*	1	1	0	0	0	0	0	0	0
Planctomycetes	*Planctomycetaceae*	1	1	2	2	1	2	4	3	3
Planctomycetes	*Candidatus Brocadiaceae*	1	1	0	0	0	0	1	1	0
Planctomycetes	*Planctomycetaceae*	1	1	2	2	1	2	4	3	3
Proteobacteria	*Rhodobacteraceae*	40	37	38	44	43	40	52	54	52
Proteobacteria	*Bradyrhizobiaceae*	20	22	21	25	24	25	24	26	25
Proteobacteria	*Rhizobiaceae*	10	12	9	13	11	11	12	12	13

Table S7 (cont.)

Phylum	Family	T1 (1)	T1 (2)	T1 (3)	T2 (1)	T2 (2)	T2 (3)	T3 (1)	T3 (2)	T3 (3)
Proteobacteria	*Rhodospirillaceae*	10	13	13	16	15	15	15	17	15
Proteobacteria	*Methylobacteriaceae*	5	5	5	5	5	5	5	6	6
Proteobacteria	*Sphingomonadaceae*	4	4	3	3	3	3	4	4	3
Proteobacteria	*Methylocystaceae*	3	2	3	3	3	3	3	3	3
Proteobacteria	*Phyllobacteriaceae*	3	3	3	3	3	3	3	3	3
Proteobacteria	*Xanthobacteraceae*	3	2	3	3	2	3	3	3	3
Proteobacteria	*Acetobacteraceae*	3	3	2	4	4	3	4	5	5
Proteobacteria	*Beijerinckiaceae*	2	2	3	3	3	2	2	3	3
Proteobacteria	*Brucellaceae*	2	1	2	5	3	3	4	4	4
Proteobacteria	*Hyphomicrobiaceae*	2	5	5	6	6	6	7	7	7
Proteobacteria	*Caulobacteraceae*	1	1	1	1	1	1	1	1	1
Proteobacteria	*Aurantimonadaceae*	1	1	0	0	1	1	0	1	1
Proteobacteria	*Erythrobacteraceae*	1	1	1	0	0	0	1	1	1
Proteobacteria	*Burkholderiaceae*	17	20	17	19	19	18	20	21	22
Proteobacteria	*Rhodocyclaceae*	10	9	9	10	8	10	11	12	11
Proteobacteria	*Comamonadaceae*	9	10	9	11	8	10	10	11	11
Proteobacteria	*Alcaligenaceae*	8	10	9	12	11	12	12	13	12
Proteobacteria	*Neisseriaceae*	7	8	6	11	8	9	13	12	11
Proteobacteria	*Nitrosomonadaceae*	4	4	2	5	5	3	6	7	7
Proteobacteria	*Oxalobacteraceae*	3	5	4	4	4	4	5	5	4
Proteobacteria	*Hydrogenophilaceae*	2	2	2	2	2	2	2	2	2
Proteobacteria	*Desulfovibrionaceae*	6	6	4	6	4	6	7	6	7
Proteobacteria	*Myxococcaceae*	6	4	6	7	7	7	7	6	7
Proteobacteria	*Geobacteraceae*	3	3	3	3	3	3	4	4	4
Proteobacteria	*Pelobacteraceae*	2	2	2	2	2	2	2	2	2
Proteobacteria	*Polyangiaceae*	2	2	2	2	2	2	2	2	2
Proteobacteria	*Bdellovibrionaceae*	1	1	1	2	1	2	2	2	2
Proteobacteria	*Desulfobacteraceae*	1	3	3	3	1	3	4	1	4
Proteobacteria	*Desulfobulbaceae*	1	1	1	1	1	1	1	1	1
Proteobacteria	*Cystobacteraceae*	1	1	1	1	1	1	1	1	1
Proteobacteria	*Kofleriaceae*	1	1	1	1	1	1	1	1	1
Proteobacteria	*Syntrophaceae*	1	1	1	1	2	2	2	1	2
Proteobacteria	*Desulfomicrobiaceae*	0	0	0	1	0	1	1	1	1
Proteobacteria	*Syntrophobacteraceae*	0	0	0	0	0	0	1	1	1
Proteobacteria	*Campylobacteraceae*	5	4	7	8	8	7	10	12	12
Proteobacteria	*Helicobacteraceae*	3	3	3	3	2	2	4	5	3
Proteobacteria	*Enterobacteriaceae*	44	45	40	51	44	45	53	57	55
Proteobacteria	*Pseudomonadaceae*	23	28	25	34	28	30	31	37	34
Proteobacteria	*Pasteurellaceae*	9	9	8	13	12	11	13	14	13
Proteobacteria	*Shewanellaceae*	6	6	5	9	9	7	6	6	5
Proteobacteria	*Halomonadaceae*	6	5	5	7	6	7	7	6	7
Proteobacteria	*Chromatiaceae*	5	4	5	5	5	4	6	6	5
Proteobacteria	*Vibrionaceae*	5	6	5	5	5	4	10	12	11
Proteobacteria	*Methylococcaceae*	4	5	5	5	5	5	5	6	6
Proteobacteria	*Xanthomonadaceae*	4	4	5	5	5	4	5	4	5
Proteobacteria	*Alteromonadaceae*	3	3	2	4	3	2	5	4	5
Proteobacteria	*Ectothiorhodospiraceae*	2	2	2	3	3	3	3	3	3

Table S7 (cont.)

Phylum	Family	T1 (1)	T1 (2)	T1 (3)	T2 (1)	T2 (2)	T2 (3)	T3 (1)	T3 (2)	T3 (3)
Proteobacteria	*Moraxellaceae*	2	2	1	2	1	2	2	4	4
Proteobacteria	*Aeromonadaceae*	1	1	1	1	1	1	1	2	2
Proteobacteria	*Succinivibrionaceae*	1	0	1	1	1	0	1	0	1
Proteobacteria	*Cardiobacteriaceae*	1	1	0	2	2	2	2	2	2
Proteobacteria	*Oceanospirillaceae*	1	1	1	1	1	1	1	1	1
Proteobacteria	*Salinisphaeraceae*	1	2	2	1	2	2	1	1	1
Proteobacteria	*Thiotrichaceae*	1	1	1	1	1	1	2	2	1
Proteobacteria	*Colwelliaceae*	0	1	1	1	1	1	1	1	1
Proteobacteria	*Pseudoalteromonadaceae*	0	0	0	1	1	0	1	1	1
Proteobacteria	*Alcanivoracaceae*	0	0	0	1	1	1	2	2	1
Proteobacteria	*Hahellaceae*	0	1	1	1	1	1	1	1	1
Proteobacteria	*Piscirickettsiaceae*	0	0	0	0	0	0	0	0	0
Spirochaetes	*Spirochaetaceae*	4	5	5	7	7	6	6	8	8
Spirochaetes	*Spirochaetaceae*	4	5	5	7	7	6	6	8	8
Spirochaetes	*Leptospiraceae*	1	1	0	1	1	0	2	2	1
Thermodesulfobacteria	*Thermodesulfobacteriaceae*	1	1	1	1	0	1	1	1	1
Verrucomicrobia	*Opitutaceae*	0	1	1	2	2	1	2	3	3
Verrucomicrobia	*Verrucomicrobiaceae*	0	1	1	1	1	0	1	1	1
Verrucomicrobia	*Methylacidiphilaceae*	1	0	1	1	0	1	1	2	2

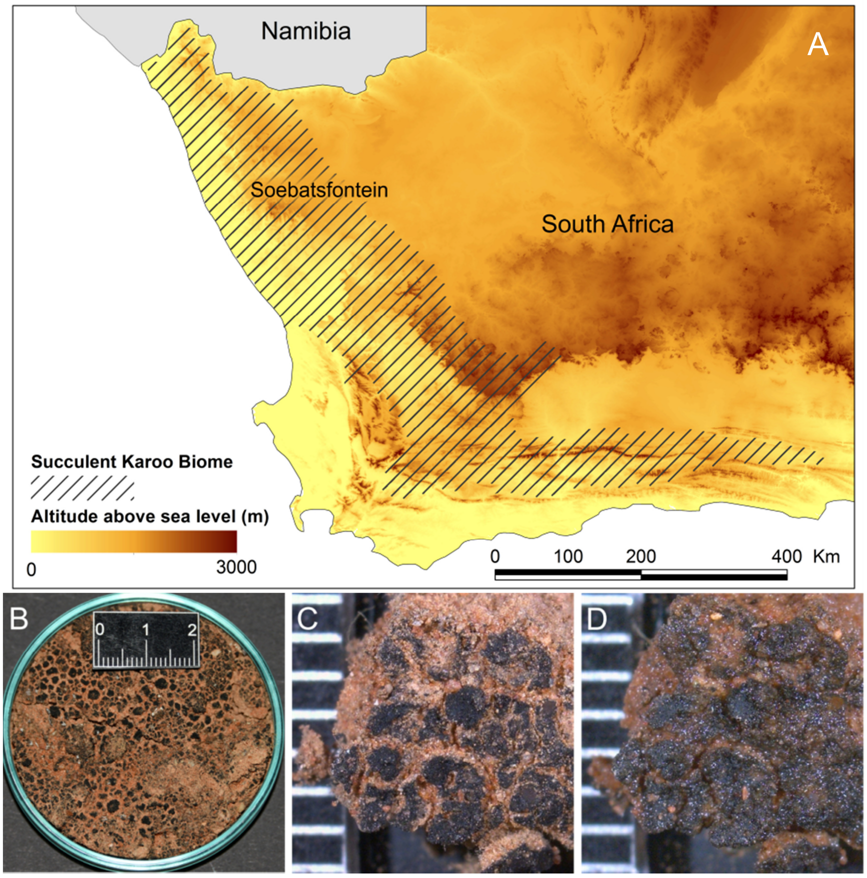

Fig. S1 Map composition presenting (A) the sampling location Soebatsfontein in South Africa and the spatial delimitation of the Succulent Karoo, (B) cyanobacteria-dominated biocrusts in an overview (scale = 2 cm), and (C, D) cyanobacteria-dominated biocrusts in close-up view taken with a stereomicroscope (scale = 7 mm; C = dry status; D = wet status).

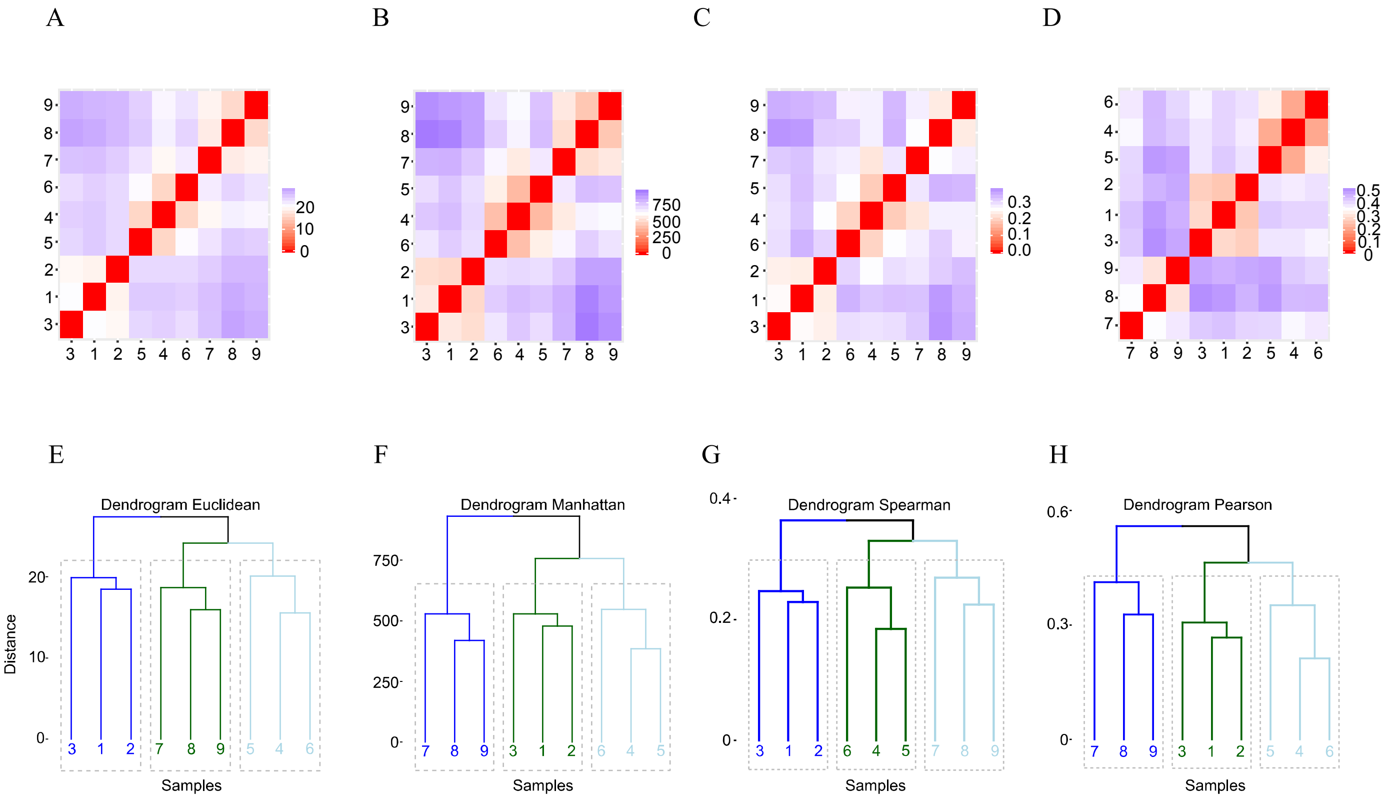

Fig. S2 Hierarchical cluster analysis based on gene expression profiles, derived from functional gene microarray analyses. Visualization of distance matrices using (A) Euclidean (B) Manhattan measures and correlation-based (C) Spearman (D) Pearson measures. Samples 1-9 are shown. Samples 1-3 were taken at stage 1, samples 4-6 at stage 2 and samples 7-9 at stage 3 during desiccation. Red: high similarity (ie: low dissimilarity), blue: low similarity. Objects of the same cluster are displayed in consecutive order. (E-H) Result of hierarchical cluster analysis. Visualization of the (dis)similarity between time points, including (E) Euclidean, (F) Manhattan distances, (G) Spearman (H) Pearson, using dendrograms. Samples that are similar to each other are combined into branches. Correlation coefficient: 0.89 (Euclidean), 0.79 (Manhattan)**,** 0.76 (Spearman), 0.88 (Pearson).

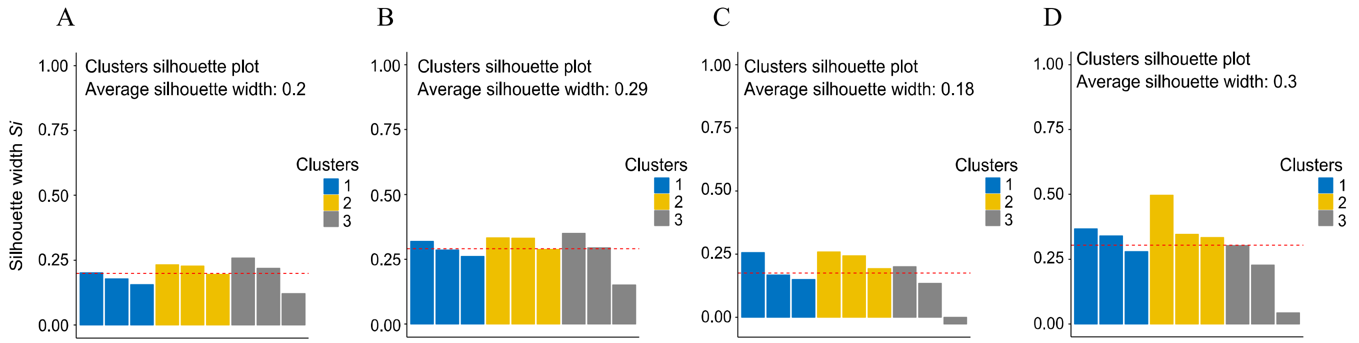

Fig. S3 Silhouette plot Cluster validation indices (Silhouette coefficient, S_i) were calculated in order to evaluate the quality of clustering algorithm results. (A) Euclidean (B) Manhattan measures and correlation-based (C) Spearman (D) Pearson measures. Silhouette coefficient (*Si*) measures how similar a sample is to the other samples in its own cluster compared to those in the other clusters. *Si* values range from 1 to -1. A value of *Si* close to 1 indicates that the sample is similar to the other objects in its group. All samples had a positive S_i indicating, that they are in the right cluster. External cluster validation using the corrected *Rand index* indicated agreement between the hierarchical clustering result and the external reference (sample design). A Rand index with the value of "1" indicated that the results of the clustering and sample structure/design were identical.

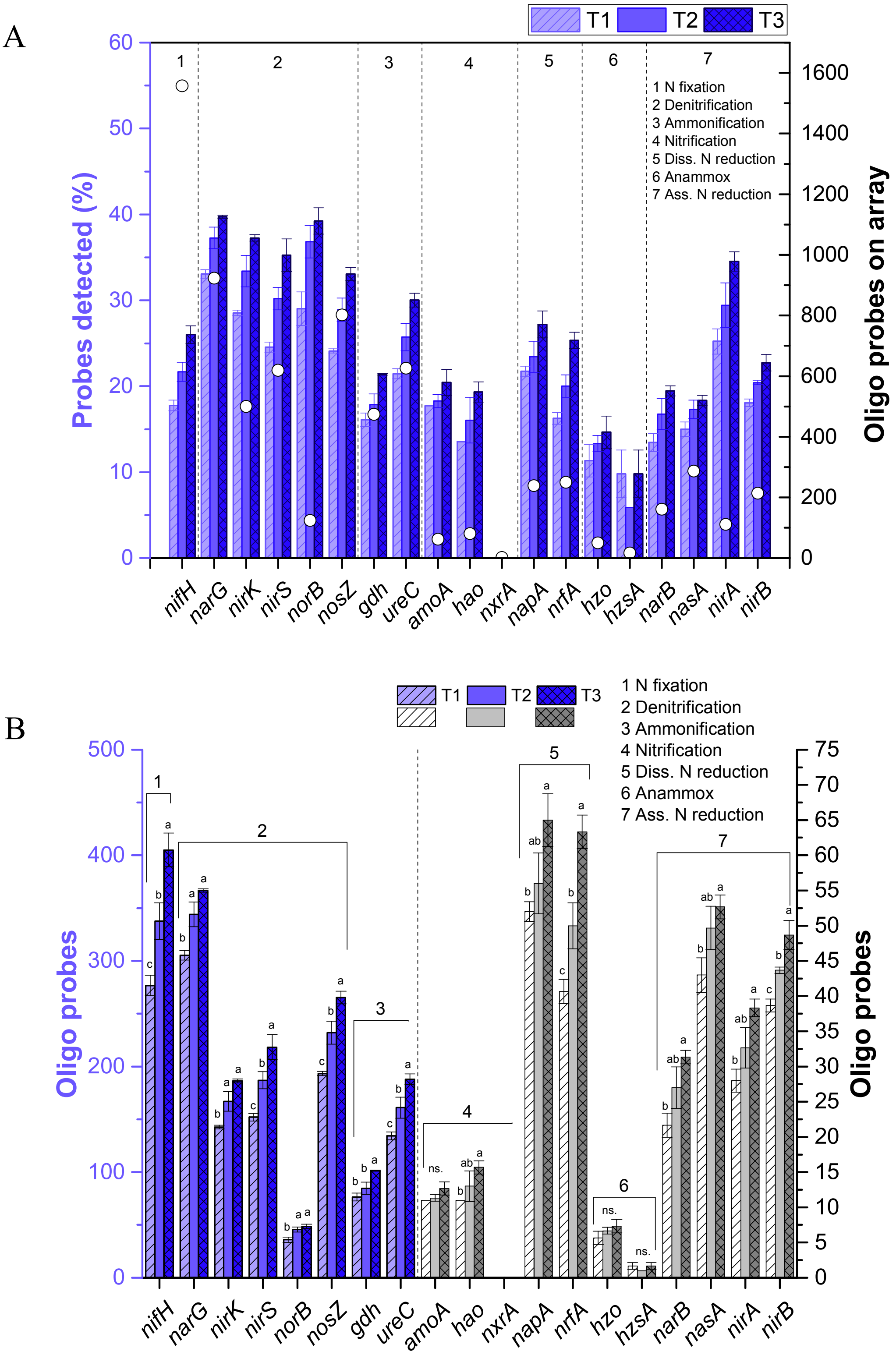

Fig. S4 Functional gene microarray. (A) Proportion of the detected probes (%) at different stages of desiccation of biocrusts. The number of probes for each gene covered on the array is shown as white dots. (B) Number of probes per gene detected at different stages of the desiccation cycle of biocrusts. Mean values of three replicates per stage during desiccation were plotted with the SDs. Differences between stages of desiccation were tested with a one-way ANOVA and labelled with lowercase letters.

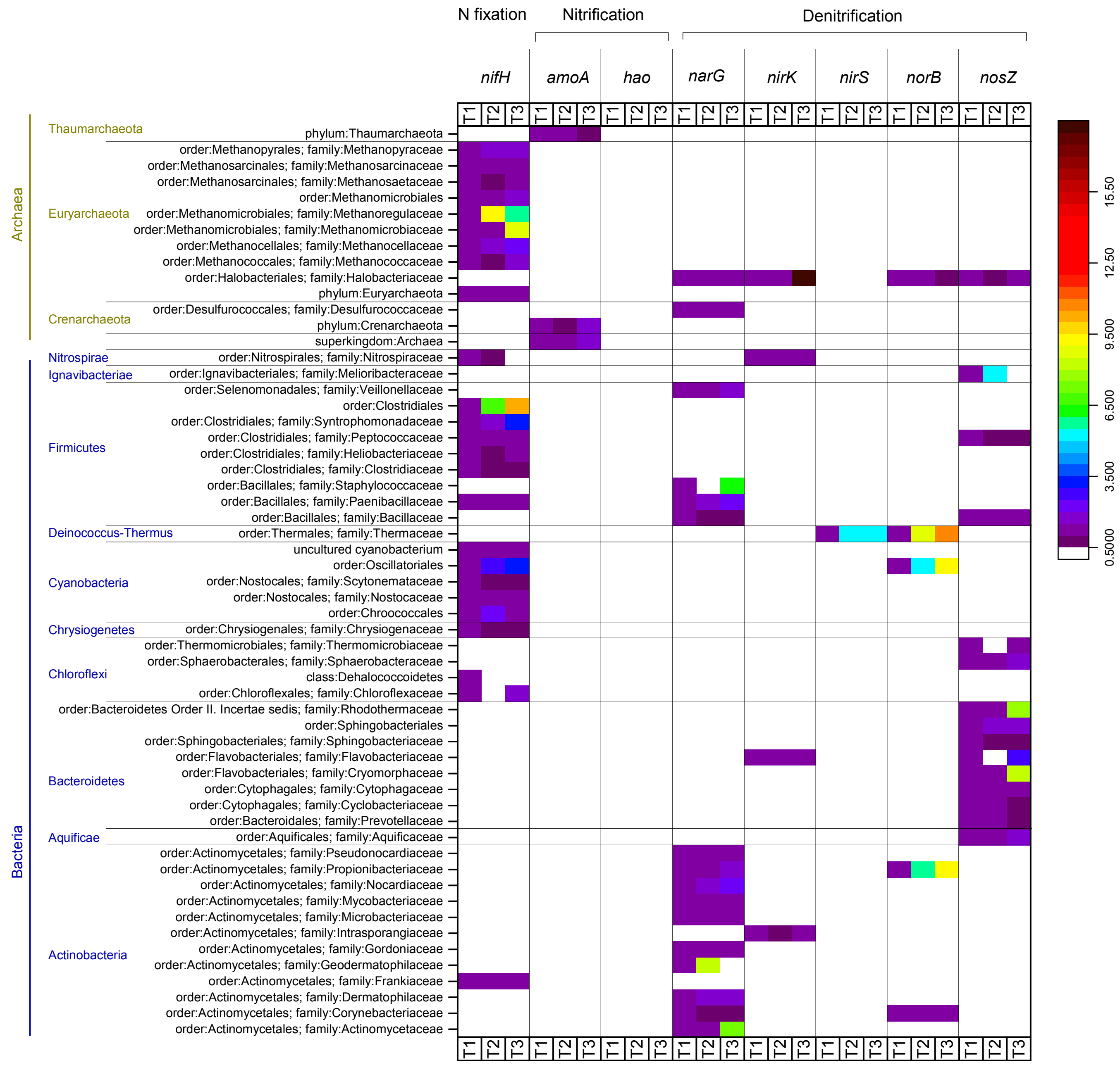

Fig. S5 Functional gene microarray. Taxa-function relationship for nitrogen-cycling genes. Mean normalized signal intensity is shown relative to T1. White colour indicates non-detected signal, while intensity of positive signals are indicated from blue (lower signal intensities) to red (higher signal intensities). Unclassified bacteria are not shown.

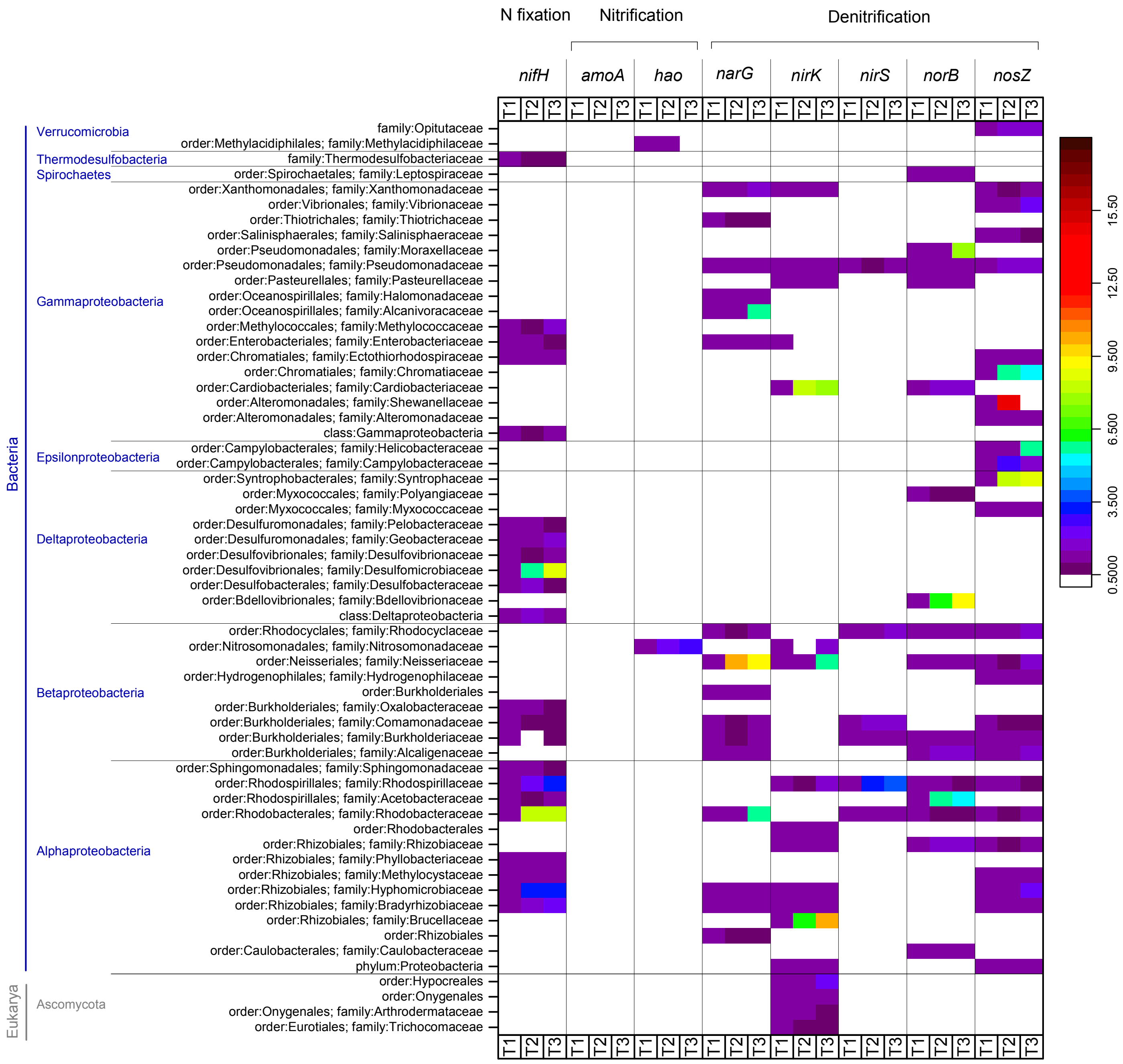

Fig. S5 (cont.) Functional gene microarray. Taxa-function relationship for nitrogen-cycling genes. Mean normalized signal intensity is shown relative to T1. White colour indicates non-detected signal, while intensity of positive signals are indicated from blue (lower signal intensities) to red (higher signal intensities). Unclassified bacteria are not shown.

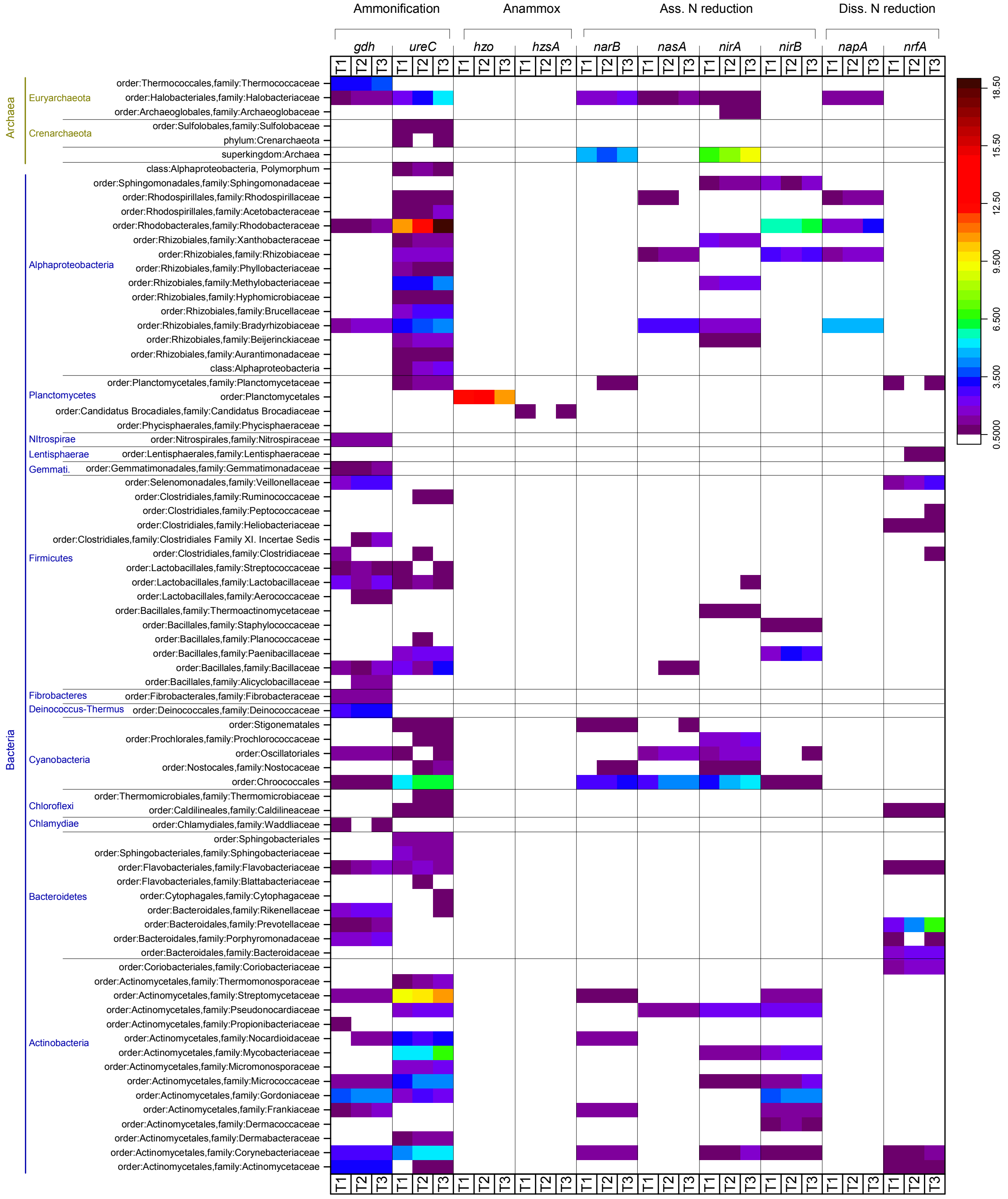

Fig. S6 Functional gene microarray. Taxa-function relationship for nitrogen-cycling genes catalysing the nitrogen-transforming processes ammonification, anammox, assimilatory and dissimilatory N reduction. Mean normalized signal intensity is shown (n = 3). White colour indicates non-detected signal, while intensity of positive signals are indicated from blue (lower signal intensities) to red (higher signal intensities). Unclassified bacteria are not shown.

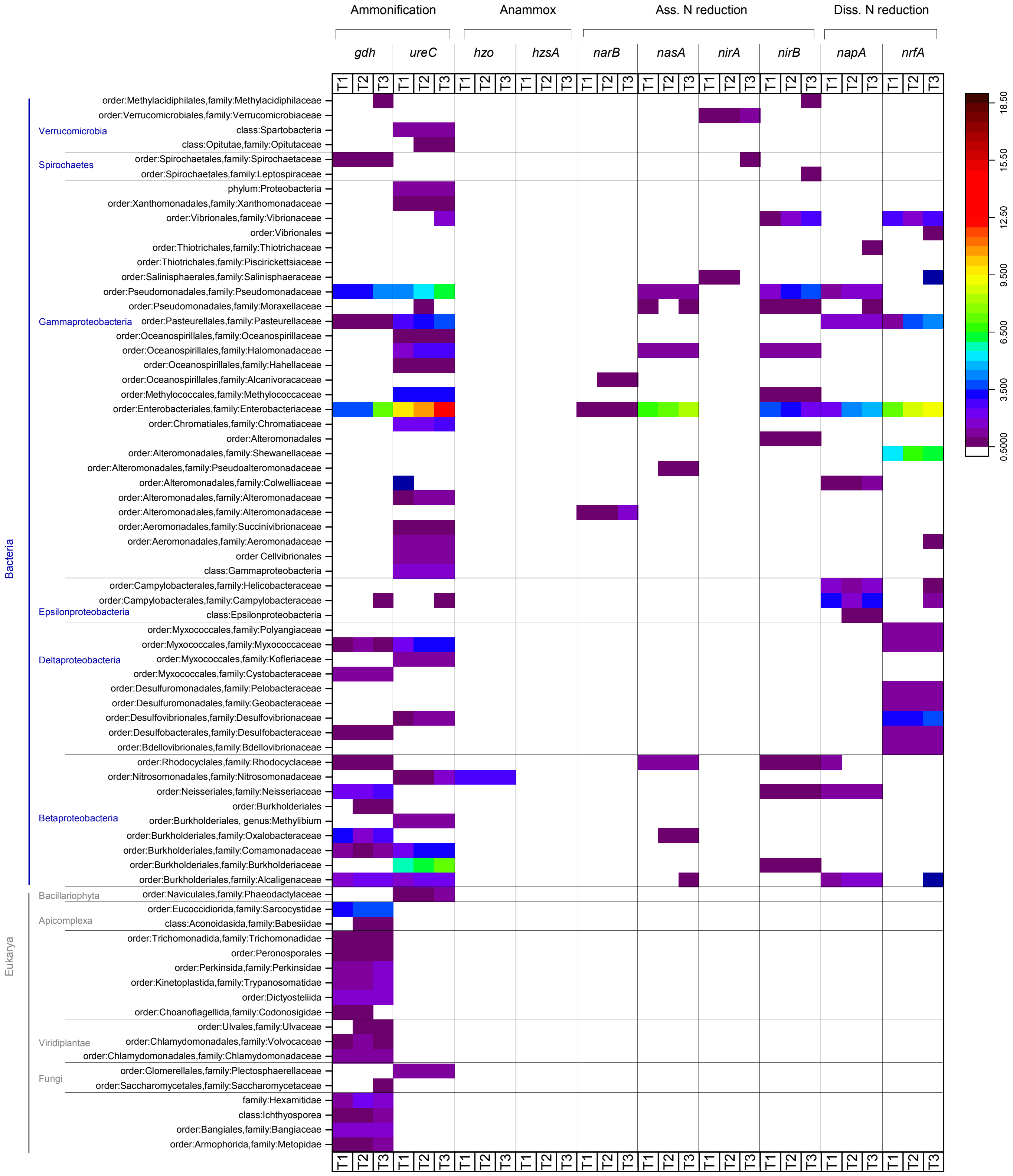

Fig. S6 (cont.) Functional gene microarray. Taxa-function relationship for nitrogen-cycling genes catalysing the nitrogen-transforming processes ammonification, anammox, assimilatory and dissimilatory N reduction. Mean normalized signal intensity is shown (n = 3). White colour indicates non-detected signal, while intensity of positive signals are indicated from blue (lower signal intensities) to red (higher signal intensities). Unclassified bacteria are not shown.

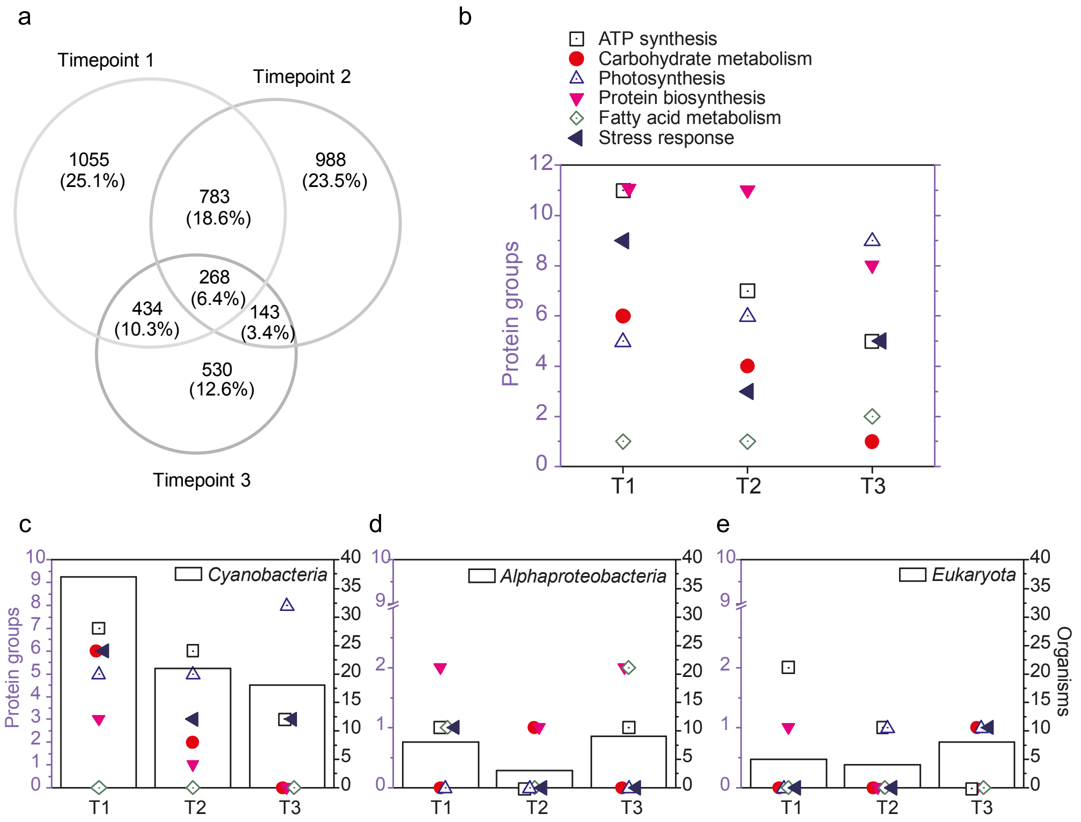

Fig. S7 Mass spectrometry-based metaproteomic data set. (a) The Venn diagram illustrates the shared and unique identified proteins for the three time stages during desiccation. (b-e) Number of protein groups, categorized into different processes based on gene ontology terms, present at the desiccation stages T1, T2, and T3 (shown as symbols). (b) Number of detected protein groups. Carbohydrate metabolisms includes the pathways glycolysis, gluconeogenesis, and pentose phosphate pathway. (c-e) Number of detected protein groups (shown as symbols) and organisms (shown as columns) within (c) Cyanobacteria, (d) Alphaproteobacteria and (e) Eukaryota.

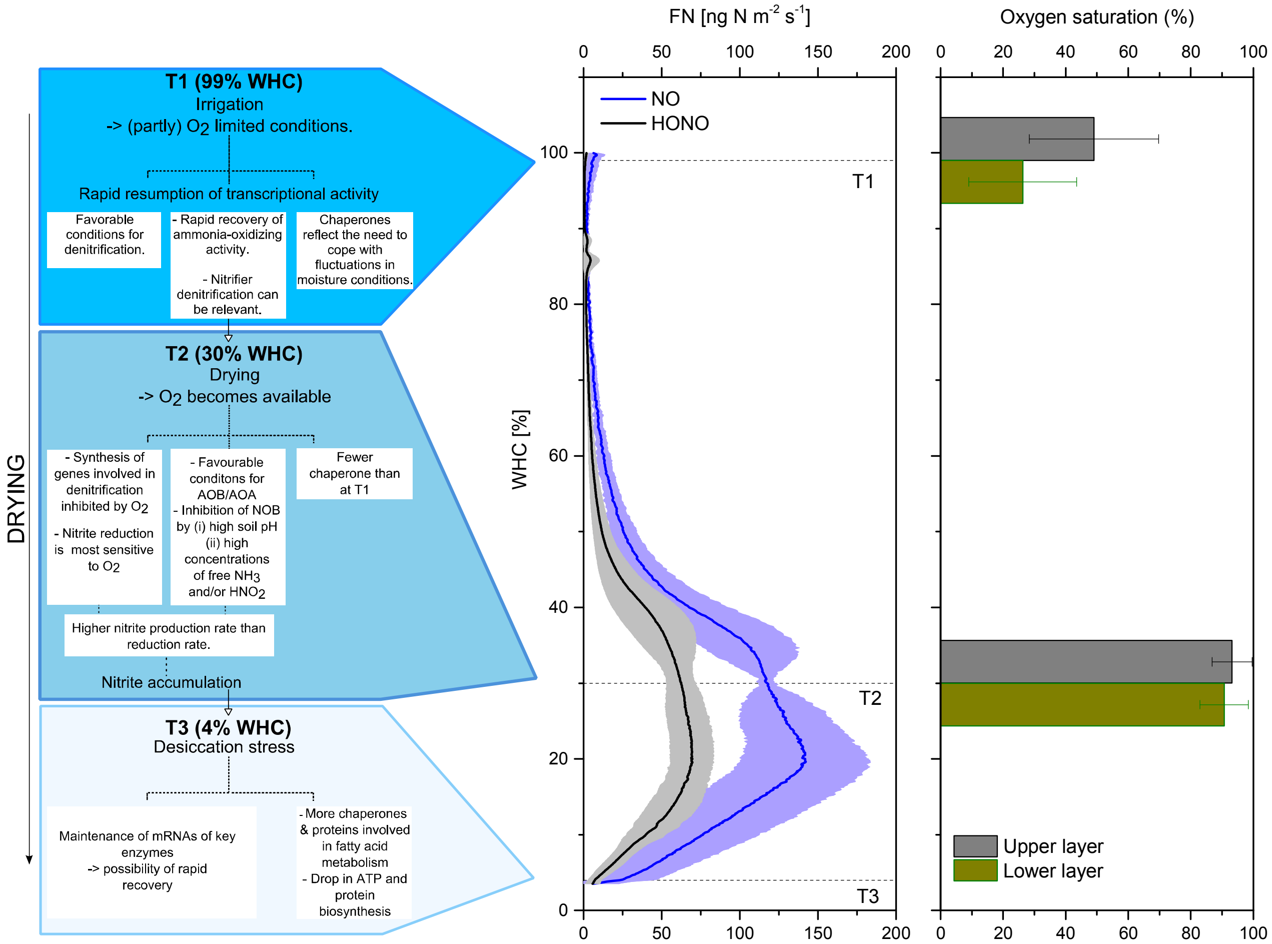

Fig. S8 Effect of soil moisture and O_2 distribution on the activity of nitrogen-transforming microorganisms within drying biocrusts

References

1. Weber B, Wu D, Tamm A, Ruckteschler N, Rodríguez-Caballero E, Steinkamp J, et al. Biological soil crusts accelerate the nitrogen cycle through large NO and HONO emissions in drylands. PNAS. 2015;112(50):15384-9.
2. Su H, Cheng YF, Oswald R, Behrendt T, Trebs I, Meixner FX, et al. Soil nitrite as a source of atmospheric HONO and OH radicals. Science. 2011;333(6049):1616-8.
3. Oswald R, Behrendt T, Ermel M, Wu D, Su H, Cheng Y, et al. HONO emissions from soil bacteria as a major source of atmospheric reactive nitrogen. Science. 2013;341(6151):1233-5.
4. Oswald R, Ermel M, Hens K, Novelli A, Ouwersloot HG, Paasonen P, et al. A comparison of HONO budgets for two measurement heights at a field station within the boreal forest in Finland. Atmos Chem Phys. 2015;15(2):799-813.
5. Wu DM, Kampf CJ, Poschl U, Oswald R, Cui JF, Ermel M, et al. Novel Tracer Method To Measure Isotopic Labeled Gas-Phase Nitrous Acid ((HONO)-N-15) in Biogeochemical Studies. Environ Sci Technol. 2014;48(14):8021-7.
6. Meusel H, Tamm A, Kuhn U, Wu D, Leifke AL, Fiedler S, et al. Emission of nitrous acid from soil and biological soil crusts represents an important source of HONO in the remote atmosphere in Cyprus. Atmos Chem Phys. 2018;18(2):799-813.
7. Heland J, Kleffmann J, Kurtenbach R, Wiesen P. A new instrument to measure gaseous nitrous acid (HONO) in the atmosphere. Environ Sci Technol. 2001;35(15):3207-12.
8. Kleffmann J, Heland J, Kurtenbach R, Lorzer J, Wiesen P. A new instrument (LOPAP) for the detection of nitrous acid (HONO). Sci Pollut Res. 2002;9(4):48-54.
9. Eickhorst T, Tippkötter R. Improved detection of soil microorganisms using fluorescence in situ hybridization (FISH) and catalyzed reporter deposition (CARD-FISH). Soil Biology and Biochemistry. 2008;40(7):1883-91.
10. Schmidt H, Eickhorst T, Tippkötter R. Evaluation of tyramide solutions for an improved detection and enumeration of single microbial cells in soil by CARD-FISH. J Microbiol Methods. 2012;91(3):399-405.
11. Schmidt H, Eickhorst T. Detection and quantification of native microbial populations on soil-grown rice roots by catalyzed reporter deposition-fluorescence *in situ* hybridization. FEMS Microbiol Ecol. 2014;87(2):390-402.
12. Carpenter B, Gelman A, Hoffman MD, Lee D, Goodrich B, Betancourt M, et al. Stan: A probabilistic programming language. J Stat Softw. 2017;76(1):1-32.
13. Bürkner P-C. brms: An R package for Bayesian multilevel models using Stan. J Stat Softw. 2017;80(1):1-28.
14. R Core Development Team. R: A language and environment for statistical computing. R Foundation for Statistical Computing, Vienna, Austria. Available online at https://www.R-project.org/. 2020.
15. Van Nostrand J, He Z, Zhou J. Use of functional gene arrays for elucidating *in situ* biodegradation. Front Microbiol. 2012;3:339.
16. Van Nostrand JD, Yin H, Wu L, Yuan T, Zhou J. Hybridization of environmental microbial community nucleic acids by GeoChip. Methods Mol Biol. 2016;1399:183-96.
17. Quackenbush J. Microarray data normalization and transformation. Nature Genetics. 2002;32:496–501.
18. Liang Y, He Z, Wu L, Deng Y, Li G, Zhou J. Development of a common oligonucleotide reference standard for microarray data normalization and comparison across different microbial communities. Appl Environ Microbiol. 2010;76(4):1088-94.
19. Sánchez A, Ruíz de Villagra MC. A tutorial review of microarray data analysis. 2008.
20. Tarca AL, Romero R, Draghici S. Analysis of microarray experiments of gene expression profiling. American Journal of Obstetrics & Gynecology. 2006;195(2):373-88.
21. Ritchie ME, Phipson B, Wu D, Hu Y, Law CW, Shi W, et al. *limma* powers differential expression analyses for RNA-sequencing and microarray studies. Nucleic Acids Res. 2015;43(7):e47.

22. Phipson B, Lee S, Majewski IJ, Alexander WS, Smyth GK. Robust hyperparameter estimation protects against hypervariable genes and improves power to detect differential expression. Ann Appl Stat. 2016;10(2):946-63.
23. Kassambara A. Practical Guide to Cluster Analysis in R: Unsupervised Machine Learning: STHDA; 2017.
24. Liu F, Lai S, Reinmuth-Selzle K, Scheel JF, Fröhlich-Nowoisky J, Després VR, et al. Metaproteomic analysis of atmospheric aerosol samples. Anal Bioana Chem. 2016;408(23):6337-48.
25. Cox J, Mann M. MaxQuant enables high peptide identification rates, individualized ppb-range mass accuracies and proteome-wide protein quantification. Nat Biotechnol. 2008;26(12):1367–72.
26. Amann RI, Binder BJ, Olson RJ, Chisholm SW, Devereux R, Stahl DA. Combination of 16S rRNA-targeted oligonucleotide probes with flow cytometry for analyzing mixed microbial populations. Appl Environ Microb. 1990;56(6):1919-25.
27. Daims H, Brühl A, Amann R, Schleifer K-H, Wagner M. The domain-specific probe EUB338 is insufficient for the detection of all bacteria: Development and evaluation of a more comprehensive probe set. Syst Appl Microbiol. 1999;22(3):434-44.
28. Burggraf S, Mayer T, Amann R, Schadhauser S, Woese CR, Stetter KO. Identifying members of the domain Archaea with rRNA-targeted oligonucleotide probes. Appl Environ Microb. 1994;60(9):3112-9.
29. Daims H, Nielsen JL, Nielsen PH, Schleifer KH, Wagner M. In situ characterization of *nitrospira*-like nitrite-oxidizing bacteria active in wastewater treatment plants. Appl Environ Microb. 2001;67(11):5273-84.
30. Daims H, Lücker S, Le Paslier D, Wagner M. Diversity, environmental genomics, and ecophysiology of nitrite-oxidizing bacteria. In: Ward BB, Arp DJ, Klotz MG, editors. Nitrification2011.
31. Mobarry BK, Wagner M, Urbain V, Rittmann BE, Stahl DA. Phylogenetic probes for analyzing abundance and spatial organization of nitrifying bacteria. Appl Environ Microb. 1996;62(6):2156-62.
32. Wallner G, Amann R, Beisker W. Optimizing fluorescent in situ hybridization with rRNA-targeted oligonucleotide probes for flow cytometric identification of microorganisms. Cytometry. 1993;14(2):136-43.
